# Characterization of Calcium‐ and Strontium‐Polyphosphate Particles Toward Drug Delivery into Articular Cartilage

**DOI:** 10.1002/mabi.202300345

**Published:** 2023-10-18

**Authors:** Jordan Nhan, Nicolas Strebel, Khushnouma Virah Sawmy, Jordan Yin, Jean‐Philippe St‐Pierre

**Affiliations:** ^1^ Department of Chemical and Biological Engineering Faculty of Engineering University of Ottawa 161 Louis‐Pasteur Pvt. Ottawa Ontario K1N 6N5 Canada

**Keywords:** articular cartilage, chondrocytes, drug delivery, inorganic polyphosphate, nanoparticles

## Abstract

Drug delivery into articular cartilage poses many challenges due in part to its lack of vasculature. While intra‐articular injections are effective for the local administration of drugs, small molecules are rapidly cleared from the synovial fluid. As such, there is a need to develop effective drug delivery strategies to improve the residence times of bioactive molecules in the joint and elicit a sustained therapeutic effect. In this study, calcium‐ and strontium‐polyphosphate particles are synthesized and characterized as potential drug carriers into articular cartilage. Physicochemical characterization reveals that the particles exhibit a spherical morphology, have a negative zeta potential, and are nanoscale in size. Biological characterization in chondrocytes confirms cellular uptake of the particles and demonstrates both size and concentration‐dependent cytotoxicity at high concentrations. Furthermore, treatment of chondrocytes with these particles results in a reduction in cell proliferation and metabolic activity, confirming biological effects. Finally, incubation with cartilage tissue explants suggests successful uptake, despite the particles exhibiting a negative surface charge. Therefore, from the results of this study, these polyphosphate‐based particles have potential as a drug carrier into articular cartilage and warrant further development.

## Graphical abstract

Polyphosphate is ionically complexed with cations to produce particles. These polyphosphate‐based particles exhibit a relatively spherical morphology and a negative surface charge. Treatment of chondrocytes with the particles indicate low cytotoxicity and confirmed bioactivity with a decrease in both metabolic activity and cell proliferation. Lastly, these particles successfully penetrated cartilage explants, demonstrating the potential for drug delivery applications into articular cartilage.

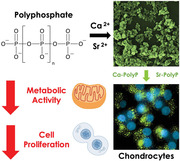

## 1 Introduction

Osteoarthritis (OA) is a degenerative joint disease characterized by the progressive loss of articular cartilage, amongst changes to other tissues in the joint. The pathogenesis of OA is complex and comprises multiple underlying risk factors and pathways leading to its development.^[^ ^1^ ^]^ Consequently, early events of OA have yet to be well established, resulting in challenges in early detection and interventions at points when disease progression may potentially be stopped or reversed. As a result, no disease‐modifying OA drugs (DMOADs) that can halt or reverse the progression of the disease have been approved for clinical use yet. Instead, current pharmacological treatments, such as analgesics and nonsteroidal anti‐inflammatory drugs (NSAIDs), focus on the management of symptoms.^[^ ^2^ ^]^

The development of DMOADs must also consider the mode of delivery for effective outcomes. As articular cartilage is avascular, systemic administration of drugs that target this tissue specifically is generally considered ineffective because it relies on entry into the joint from the capillaries of the synovium. Indeed, the endothelial lining acts as a sieve, limiting the rate of passage for larger molecules above ≈10 kDa, while diffusion into the joint is hindered by the extracellular matrix (ECM) of the synovium.^[^ ^3^ ^]^ Local administration of drugs into the joint cavity via intra‐articular injection can circumvent these mass transfer limitations and allow for increased local bioavailability of the therapeutic, while also reducing off‐target and adverse effects, and decreasing total drug costs.^[^ ^3^, ^4^ ^]^ However, maintaining the drug at therapeutic concentrations to elicit a prolonged, positive effect poses additional challenges. Small molecules are rapidly cleared by synovial capillaries, while larger macromolecules may be cleared by the lymphatic system.^[^ ^3^ ^]^ For example, the half‐life of various drugs such as NSAIDs administered via intra‐articular injection is ≈1–5 h.^[^ ^5^ ^]^ Such short residence times in the joint are likely insufficient for the treatment of a chronic condition such as OA with DMOAD candidates.

Furthermore, the dense ECM of articular cartilage, which is primarily composed of self‐assembled networks of collagens and proteoglycans, can act as a physical barrier to the entry and diffusion of drugs into the tissue. In fact, the spacing between collagen fibrils is estimated to be between 60 and 200 nm.^[^ ^6^, ^7^, ^8^ ^]^ These collagen networks entrap proteoglycan aggregates, creating a dense ECM arrangement, which presents substantial steric hindrance for the delivery of drugs into the tissue, requiring them to be nano or submicron in size.^[^ ^8^ ^]^ In addition, due to the high abundance of glycosaminoglycans (GAGs), cartilage tissue has a highly negative fixed charge, with an average fixed charged density of −170.0 mm.^[^ ^9^ ^]^ This has been proposed to act as an additional barrier of entry for some compounds as a result of electrostatic repulsive forces. While the degradation of the ECM components of cartilage in moderate to severe cases of OA will facilitate entry of drug carriers, this may not be exploited during the early stages of the disease, when the structural integrity of the tissue has not yet been substantially compromised.^[^ ^10^ ^]^ Early interventions may be key in stopping or reversing disease progression, and therefore there is a need to develop drug delivery strategies that allow for effective and sustained delivery of therapeutics into affected cartilage tissue by taking into consideration as many of these barriers as possible.

Particle‐based drug delivery systems have been of interest for cartilage tissue applications and may be broadly classified as either microparticles or nanoparticles based on the size of the carrier. Due to the larger size, particle‐based drug carriers are not as easily cleared from the synovial fluid compared to soluble drug molecules. While larger particles will be retained within the synovial fluid for a longer period of time, they may not effectively penetrate into cartilage tissue and instead rely on sustained release. Conversely, smaller particles are rapidly cleared from the joint, but have an improved capability to penetrate deeper into the cartilage tissue, allowing for a more targeted drug delivery approach and additional protection from clearance.

Polyphosphate (polyP) is an inorganic biopolymer of orthophosphate units present in both prokaryotes and eukaryotes.^[^ ^11^ ^]^ In mammalian cells and tissues, it has been found to be localized to the cytosol, nucleus, lysosomes, and mitochondria,^[^ ^11^ ^]^ hinting at a broad range of biological functions, with localization of polyP to the mitochondria and its high‐energy phosphoanhydride bonds implicating a functional role in metabolic processes. While an increased interest in this biomolecule has led to the identification of a number of its functions in mammalian cells,^[^ ^12^ ^]^ the biological roles of endogenous polyP still remain poorly understood. Previous work demonstrated that treatment of chondrocytes with soluble polyP resulted in a significant increase in ECM accumulation for in vitro‐grown cartilage tissues.^[^ ^13^ ^]^ These results identify polyP as a molecule of interest for cartilage tissue formation with potential applications in tissue engineering and as a therapeutic, which have now been supported by other studies.^[^ ^14^, ^15^, ^16^, ^17^ ^]^ As the biological functions of polyP are being identified, the development and characterization of polyP‐based materials to influence cell responses is a crucial step to exploiting its properties.

Herein, we synthesized calcium‐polyphosphate (Ca‐polyP) and strontium‐polyphosphate (Sr‐polyP) particles and characterized these polyP‐based particles for use in drug delivery applications in cartilage. Calcium was selected as an ionic cross‐linker given that the anabolic effects of polyP in chondrocytes have been attributed to calcium signaling,^[^ ^18^ ^]^ which suggests a synergistic effect of Ca‐polyP. Meanwhile, strontium has been demonstrated to promote anabolic pathways in chondrocytes,^[^ ^19^ ^]^ inhibit catabolic proteases and inflammatory cytokines in an OA animal model,^[^ ^20^ ^]^ and protect against cartilage loss in OA patients.^[^ ^21^ ^]^ Physicochemical characterization of the size stability, surface charge, and composition of these particles revealed new information on the organization of polyP within such materials and on the effect of substituting cross‐linking cations on colloidal stability. Treatment of chondrocytes with these particles identified that the particles are bioactive, modulating both cell proliferation and metabolic activity, with cytotoxic effects only at high concentrations. Particle uptake and retention into cartilage tissue explants was also confirmed, suggesting that negatively charged particles may penetrate cartilage relatively efficiently and could be used for drug delivery applications.

## 2 Results and Discussion

### 2.1 Physicochemical Characterization of PolyP‐Based Particles

**Figure 1:**
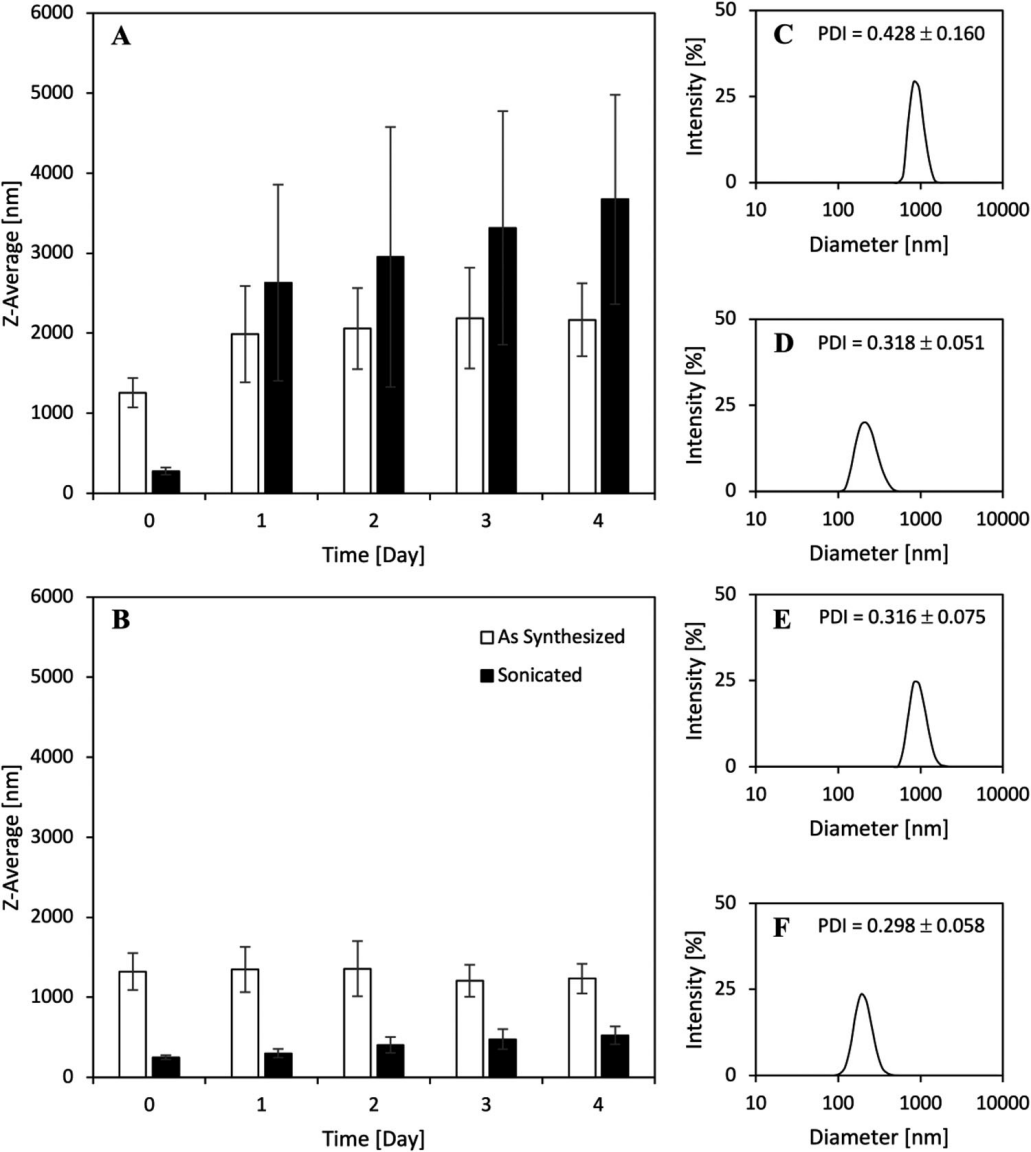
Size and stability of polyP‐based particles in diH_2_O. PolyP‐based particle size was measured by DLS in diH_2_O for A) Ca‐polyP and B) Sr‐polyP particles. Representative PSD and the average PDI for C) Ca‐polyP particles as synthesized or D) sonicated and E) Sr‐polyP particles as synthesized or F) sonicated as measured at day 0 are indicated. Data are presented as averages ± standard deviation for *n* ≥ 4 experiments.

To assess the size stability of the synthesized Ca‐polyP and Sr‐polyP particles, both as synthesized and sonicated particles were stored in deionized water (diH_2_O), and dynamic light scattering (DLS) was performed daily over the course of 4 days (**Figure** **1**A,B). Probe sonication successfully reduced the average hydrodynamic diameter of Ca‐polyP particle agglomerates from 1257 ± 184 nm to 277.7 ± 45.0 nm (*p* < 0.0001) and Sr‐polyP particle agglomerates from 1322 ± 231 nm to 250.7 ± 25.9 nm (*p* < 0.0001), reducing the size of particles (or agglomerates) to a submicron scale. Interestingly, particles in the range of 100–300 nm in size have been reported to selectively enter OA cartilage but not healthy cartilage, suggesting that sonication yields particles with adequate sizes for cartilage uptake.^[^ ^22^, ^23^ ^]^ As can be seen in the particle size distributions (PSD) (Figure 1C–F), the population exhibits a unimodal distribution that is moderately polydisperse based on the average polydispersity index (PDI).^[^ ^24^, ^25^ ^]^ Furthermore, as the PDI values for the sonicated Ca‐polyP and Sr‐polyP are below 0.4, the particle population is considered to be sufficiently uniform in size for biomedical applications.^[^ ^26^ ^]^

While the initial size of Ca‐polyP and Sr‐polyP are quite similar for both the as synthesized and sonicated conditions, Ca‐polyP exhibits much poorer size stability compared to Sr‐polyP. Comparing the particles as synthesized, Ca‐polyP particles increase in size after 1 day of incubation (*p* < 0.05), and the particle size remains quite stable thereafter, with a 1.7‐fold increase by day 4. Meanwhile, Sr‐polyP particles remain similar in size throughout the entire 4 days, with no significant differences in the size between all time points. These trends in size stability are also observed for the sonicated polyP‐based particles, but to a greater degree. The sonicated Ca‐polyP particles rapidly agglomerate following sonication, with a significant increase between the hydrodynamic diameter at day 0 and day 1 (*p* < 0.01), even exceeding the size of the as synthesized Ca‐polyP particles, with an over 13‐fold increase by day 4. In contrast, Sr‐polyP particles agglomerate at a slower rate, with a statistically significant increase beginning between day 0 and day 2 (*p* < 0.01) but overall, the hydrodynamic diameter remains much smaller than the as synthesized Sr‐polyP throughout the entire 4 days, with only an over 2‐fold increase by day 4. Of note, sonication of agglomerates formed after the 4 day incubation period successfully redispersed the particles to a similar size as that observed at day 0 (data not shown), suggesting that agglomeration does not lead to strong interactions between particles.

The mechanism leading to the improved size stability of Sr‐polyP in water compared to Ca‐polyP is unknown at this point. The zeta potential of Ca‐polyP and Sr‐polyP in diH_2_O was measured to be −22.7 ± 3.2 mV and −21.5 ± 2.6 mV respectively, indicating negligible differences in the surface charge. Therefore, colloidal stability is likely not attributed to differences in electrostatic repulsive forces between individual particles. While calcium and strontium ions contain many similarities, their chemistry and interactions with other molecules can differ drastically. For example, the ionic radii of strontium ions are slightly larger than calcium ions, which may affect its bonding and cross‐linking with polyP due to potential changes in the crystallinity, lattice parameters, crystal size, morphology, solubility, and stability of the material.^[^ ^27^ ^]^ Another study performed X‐ray diffraction analysis on polyP‐based scaffolds, which indicated a monoclinic crystal system in calcium polyP and a rhombohedral crystal system in strontium polyP structures.^[^ ^28^ ^]^ As crystallinity can influence the size stability of nanoparticles,^[^ ^29^ ^]^ this may be a contributing factor in the improved size stability of the Sr‐polyP particles.

**Figure 2:**
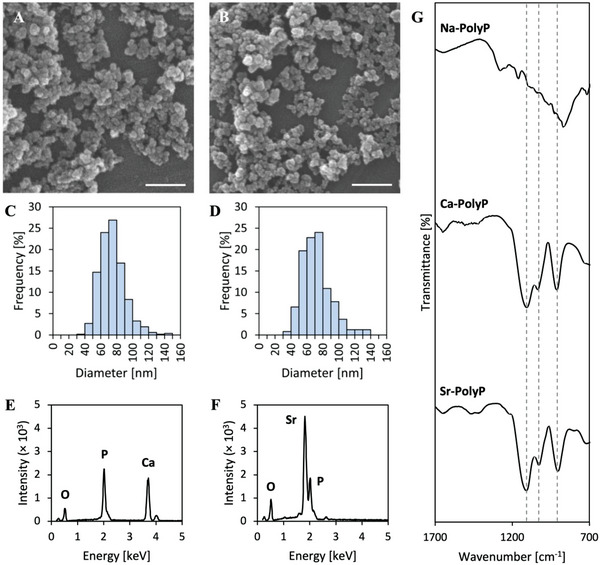
PolyP‐based particle morphology and composition. SEM images of A) Ca‐polyP and B) Sr‐polyP particles along with PSD of C) Ca‐polyP and D) Sr‐polyP particles measured from SEM images. EDX spectra of E) Ca‐polyP and F) Sr‐polyP particles, along with G) FTIR spectra of Na‐polyP starting material and polyP‐based particles. Scale bar = 500 nm.

Scanning electron microscopy (SEM) was performed on sonicated Ca‐polyP and Sr‐polyP, which suggests that the particles exhibit a relatively spherical morphology (**Figure** **2**A,B). Particle diameters estimated from SEM have an average diameter of 74 ± 16 nm (*n* = 517) and 72 ± 18 nm (*n* = 487) for Ca‐polyP and Sr‐polyP respectively (Figure 2C,D). Given that the individual particles are nanoscale in size, these particles may be suitable for drug delivery applications in cartilage tissue, even in the early stages of OA development. The differences between DLS and SEM measurements may be explained by the fact that the scattering intensity measured by DLS is proportional to the square of the particle size, such that the z‐average skews toward larger particles; however, it is also possible that the current sonication protocol cannot effectively break up agglomerates into individual particles or that the particles are stable as small aggregates in solution following sonication, leading to the larger sizes observed in DLS.

Energy dispersive X‐ray spectroscopy (EDX) was also performed on the particles (Figure 2E,F), confirming that the particles are composed of calcium or strontium, phosphorus, and oxygen. This also confirms that the particle preparation was mostly clean, with minimal impurities of sodium and chloride ions present during the synthesis. EDX suggests a ≈1:1 atomic ratio of Ca:P and Sr:P for Ca‐polyP and Sr‐polyP respectively. The theoretical molar ratio of divalent cations to phosphorus in such polyP materials for a pure precipitate is 0.50. The Ca/P ratio in a calcium polyP scaffold was previously reported to be 0.45,^[^ ^15^ ^]^ while another calcium phosphate‐based material often used for drug delivery, hydroxyapatite, is characterized by a much higher theoretical Ca/P ratio of 1.67. To confirm the presence of polyP, Fourier‐transform infrared (FTIR) spectroscopy was performed on dried Ca‐polyP and Sr‐polyP particles and compared to sodium‐polyphosphate (Na‐polyP) (Figure 2G). PolyP exhibits a spectral shift to two main peaks at ≈908 and 1107 cm^−1^ in both Ca‐polyP and Sr‐polyP due to the ionic cross‐linking of polyP with the divalent cations.^[^ ^16^ ^]^ Similar FTIR spectra were obtained for Ca‐polyP^[^ ^16^, ^30^ ^]^ and Sr‐polyP^[^ ^31^ ^]^ as previously reported by others. Meanwhile, a phosphate peak at ≈1028 cm^−1^ is also observed in the spectra for both Ca‐polyP and Sr‐polyP, which may suggest partial hydrolysis of polyP into free phosphate groups or incorporation of orthophosphate from the initial polyP stocks. This could also explain the intermediate Ca/P or Sr/P ratio observed with these particles. Taken together, EDX and FTIR results confirm the presence of polyP in the particles.

**Figure 3:**
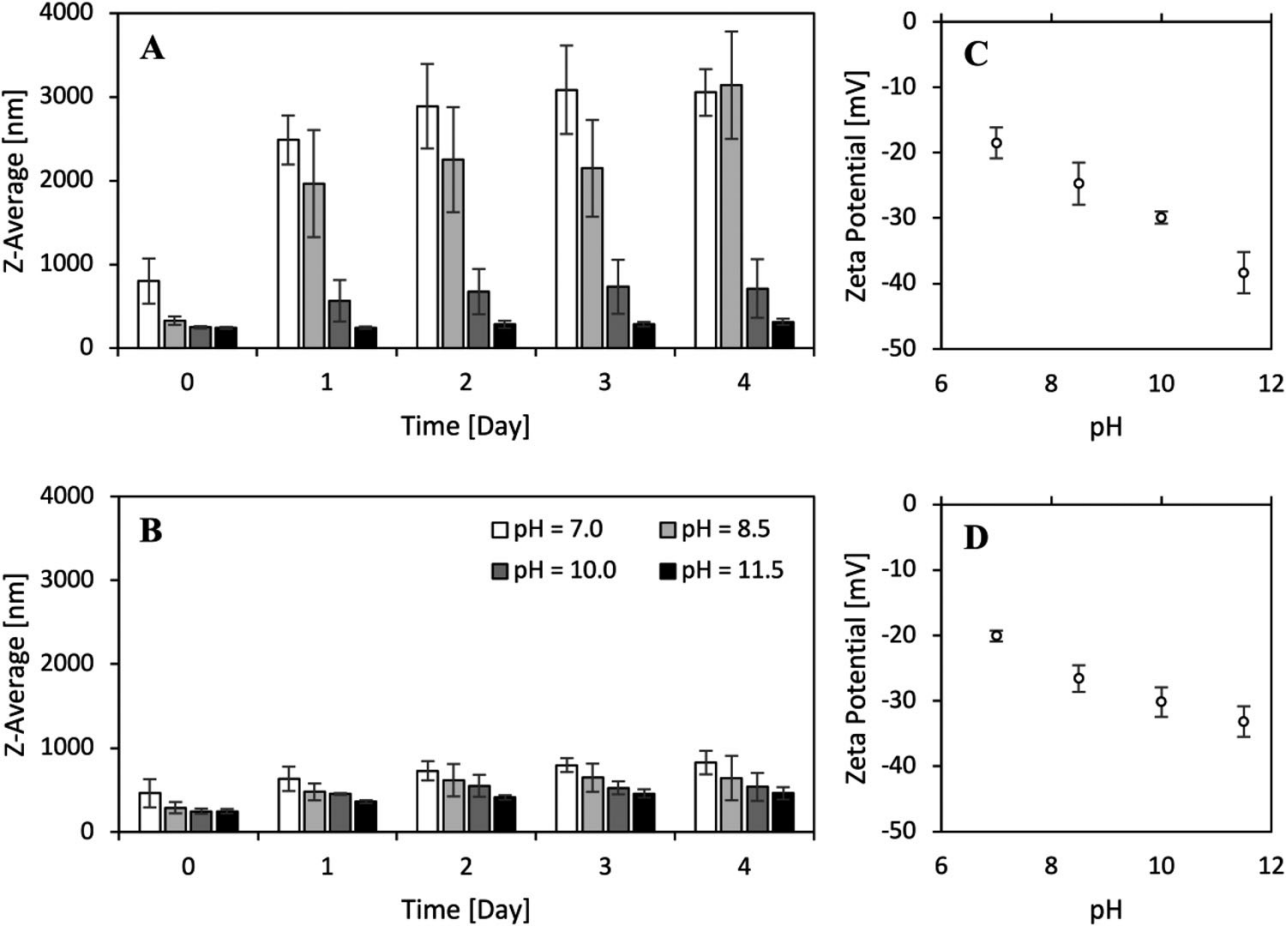
Effect of pH on polyP‐based particle size and surface charge. Sonicated polyP‐based particle size was measured by DLS in 8 mm Tris for A) Ca‐polyP and B) Sr‐polyP particles at varying pH values. Zeta potential was also measured for sonicated C) Ca‐polyP and D) Sr‐polyP particles at varying pH. Data are presented as averages ± standard deviation for *n* = 3 experiments.

As polyP is a highly ionizable inorganic polymer, the effect of pH on the size stability and surface charge was explored. Both Ca‐polyP and Sr‐polyP were sonicated in 8 mm Tris at a pH between 7.0 and 11.5, and DLS was performed over 4 days to assess size stability (**Figure** **3**A,B). The particles were maintained in 8 mm Tris to ensure that the pH remained stable throughout the entire experiment. As observed, pH has a notable effect on size stability. Zeta potential measurements were also performed, and as the pH is increased, the zeta potential measured increasingly becomes more negative (Figure 3C,D). Interestingly, the effect of pH is much more notable for Ca‐polyP than Sr‐polyP. This may be explained by the increased propensity of Ca‐polyP particles to agglomerate compared to Sr‐polyP. Furthermore, it is noted that Ca‐polyP particles generally exhibit larger changes in zeta potential between pH conditions than Sr‐polyP, with these increased electrostatic repulsive forces having a larger influence on the size stability of Ca‐polyP.

Each phosphate subunit within a polyP chain contains a strongly acidic hydrogen (p*K*_a_ 0–3) while the terminal phosphates of polyP each contain one weakly acidic hydrogen (p*K*_a_ 7–9).^[^ ^32^ ^]^ It is expected that the degree of ionization for each strongly acidic hydrogen would not change due to complete ionization at the tested pH range of 7.0–11.5, as estimated by the Henderson‐Hasselbalch equation (Table S1, Supporting Information). However, pH would have a large effect on the ionization status of the weakly acidic hydrogen present in the end‐groups of polyP molecules (Table S1, Supporting Information). It is thought that the increasingly negative zeta potential at more alkaline pH may be attributed to increased ionization of the end‐groups, which may suggest that the end‐groups are enriched at the surface of the particles, possibly due to the preferred orientation of the polyP molecules when cross‐linked with the divalent cations. As a result, the end‐groups may have a great contribution toward the surface charge of the particles.

**Figure 4:**
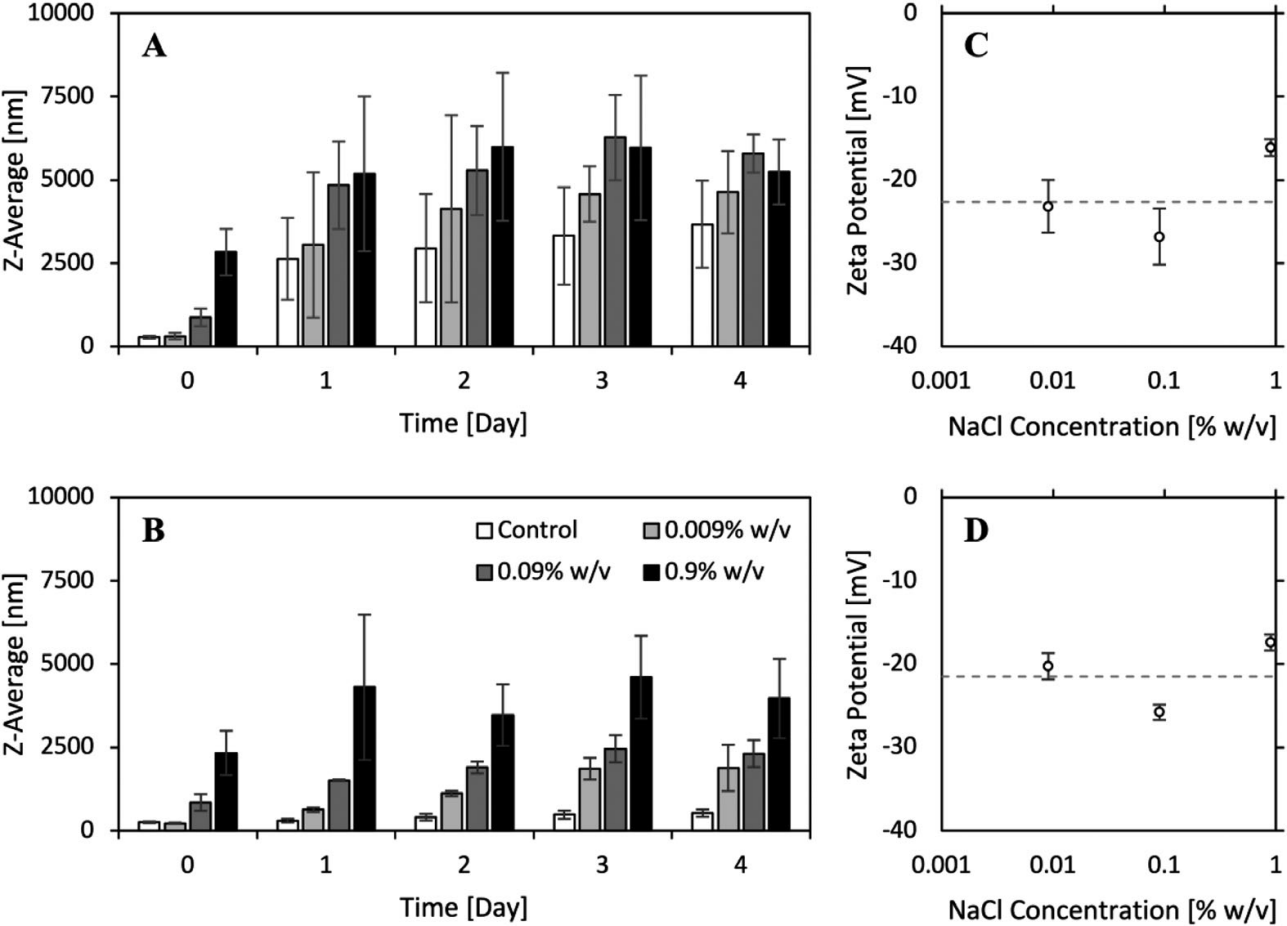
Effect of ionic strength on polyP‐based particle size and surface charge. Sonicated polyP‐based particle size was measured by DLS in varying concentrations of sodium chloride for A) Ca‐polyP and B) Sr‐polyP particles. Zeta potential was also measured for sonicated C) Ca‐polyP and D) Sr‐polyP particles at varying osmolarities. The dotted lines represent the average zeta potential measured in diH_2_O. Data are presented as averages ± standard deviation for *n* = 3 experiments.

The effect of ionic strength on the particle size stability was also evaluated. Both Ca‐polyP and Sr‐polyP were incubated in varying concentrations of NaCl, and DLS was performed to assess size stability over time (**Figure** **4**A,B). The concentration of 0.9% NaCl is at a physiological osmolarity and was selected as the highest salt concentration. It is observed that increasing salt concentration results in a decrease in particle size stability, which has been similarly reported by others.^[^ ^33^, ^34^, ^35^ ^]^ An increase in ionic strength results in the screening of electrostatic interactions, resulting in particle agglomeration. Zeta potential was also measured, with no clear trends observed (Figure 4C,D). However, this screening effect results in a more neutral zeta potential observed at 0.9% NaCl for both Ca‐polyP and Sr‐polyP. It does appear that the zeta potential has a parabolic trend as a function of salt concentration, in which a potential minimum is present at ∼0.09% NaCl for both Ca‐polyP and Sr‐polyP. Many studies have also reported observing an ionic strength‐dependent maximum or minimum zeta potential,^[^ ^36^, ^37^, ^38^, ^39^ ^]^ and this phenomenon is system‐dependent.^[^ ^40^ ^]^

**Figure 5:**
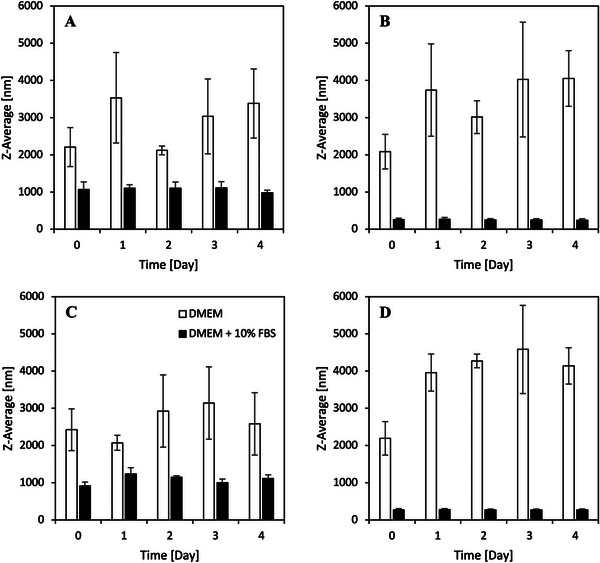
Size of polyP‐based particles in cell culture medium. PolyP‐based particle size was measured by DLS in low glucose DMEM with or without 10% FBS supplementation for A) Ca‐polyP as synthesized or B) sonicated and C) Sr‐polyP as synthesized or D) sonicated, incubated at 37 °C. Data are presented as averages ± standard deviation for *n* = 3 experiments.

Next, the particles were characterized in Dulbecco's Modified Eagle's Medium (DMEM) with or without 10% fetal bovine serum (FBS) supplementation to investigate their stability in a solution that better recapitulates a physiological environment. The particles were incubated in these media at 37 °C, and DLS was performed over 4 days (**Figure** **5**). Evidently, all particles agglomerated immediately when added to DMEM; however, particles added to DMEM with 10% FBS exhibited high colloidal stability, with the particle size remaining relatively constant throughout the 4 days. It has been well established that proteins readily adsorb onto the surfaces of particles to form a protein corona, which is dependent on the particle composition, size, shape, surface charge, and chemistry, as well as environmental factors.^[^ ^41^, ^42^, ^43^ ^]^ Similar studies have also demonstrated an improved colloidal stability in serum‐supplemented media.^[^ ^44^, ^45^, ^46^, ^47^, ^48^, ^49^, ^50^ ^]^ Adsorption of proteins typically leads to steric stabilization by preventing attractive van der Waals forces between particles through osmotic pressure and elastic recoil effects.^[^ ^51^ ^]^ Zeta potential measurements were also compared in the different media (**Table** **1**). As is also observed in 0.9% NaCl solution, there is a decrease in the magnitude of the zeta potential in phosphate‐buffered saline (PBS) and DMEM as compared to diH_2_O, which agrees with observations in previous reports with various cell culture media.^[^ ^50^, ^52^, ^53^ ^]^ Others have reported that particles with a negative zeta potential exhibit an appreciable reduction in the absolute of the zeta potential following the addition of serum proteins,^[^ ^44^, ^54^, ^55^, ^56^ ^]^ while only a slight decrease was observed from our results. This relatively small effect of the presence of serum proteins was also reported by at least one other group.^[^ ^50^ ^]^ Importantly, another study also corroborates our results, demonstrating that the zeta potential of Ca‐polyP particles synthesized via a similar process to that described here significantly decreases toward a more neutral surface charge in culture medium supplemented with FBS, with values comparable to those observed for our particles.^[^ ^57^ ^]^

**Table 1:** Zeta potential of sonicated polyP‐based particles in various media.

Medium	Zeta Potential [mV]	
	Ca‐PolyP	Sr‐PolyP
diH_2_O	−22.7 ± 3.2	−21.5 ± 2.6
0.9% NaCl	−16.1 ± 1.0^c^ ^)^	−17.4 ± 1.0^a^ ^)^
PBS	−13.7 ± 1.6^d^ ^)^	−14.9 ± 3.8^b^ ^)^
DMEM	−13.2 ± 2.5^d^ ^)^	−12.9 ± 0.8^c^ ^)^
DMEM + 10% FBS	−12.0 ± 0.5^d^ ^)^	−12.1 ± 1.5^c^ ^)^

Data are presented as averages ± standard deviation for *n* ≥ 3 experiments.

^a)^ *p* < 0.05;

^b)^ *p* < 0.01;

^c)^ *p* < 0.001;

^d)^ *p* < 0.0001 compared to the diH_2_O control for each condition.

### 2.2 Biological Characterization of Particles in Chondrocytes and Cartilage Tissues

**Figure 6:**
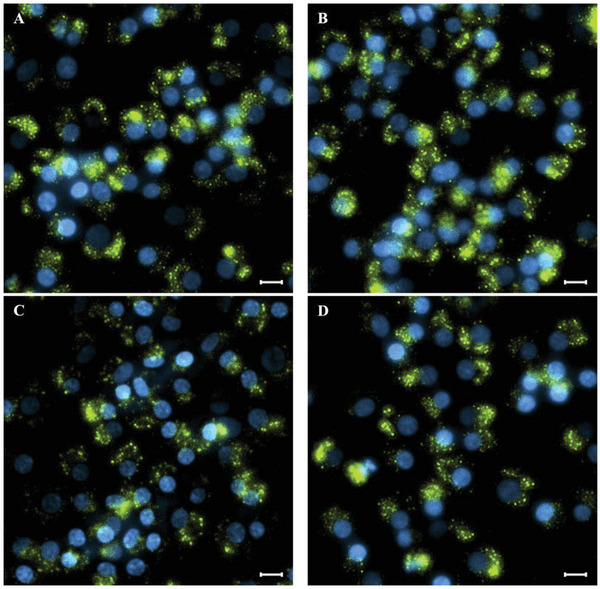
Intracellular uptake of polyP‐based particles. Primary bovine chondrocytes were treated with 100 µg mL^−1^ of DAPI‐stained A) Ca‐polyP as synthesized or B) sonicated and C) Sr‐polyP as synthesized or D) sonicated for 24 h. Representative images for each condition have both nuclei (blue) and polyP‐based particles (green) stained by DAPI. Scale bar = 20 µm.

As a next step toward evaluating the potential of these polyP‐based particles for delivery applications into the joint, their interactions with primary bovine chondrocytes were characterized. Firstly, the ability of chondrocytes to uptake these particles was tested via incubation with polyP‐based particles stained with 4',6‐diamidino‐2‐phenylindole (DAPI). To ensure that only intracellular particles were visualized, trypan blue was utilized to quench extracellular fluorescence prior to imaging, which was validated using DAPI‐stained particles alone (Figure S1, Supporting Information). Cellular uptake was confirmed through fluorescent microscopy (**Figure** **6**). As can be observed, chondrocytes were able to internalize both the as synthesized and sonicated polyP‐based particles, and no clear differences in cellular uptake were observed between conditions. It remains unclear at this point if polyP‐based particles require entry into the cells to elicit bioactivity and if the pathways by which internalization takes place influence these effects.

**Figure 7:**
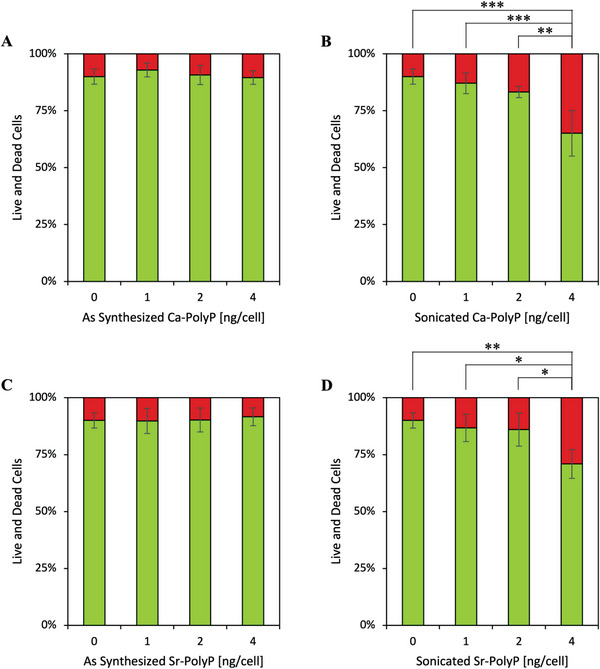
Cytotoxicity of polyP‐based particles. Primary bovine chondrocytes were treated with A) Ca‐polyP as synthesized or B) sonicated and C) Sr‐polyP as synthesized or D) sonicated at varying concentrations for 24 h. Chondrocytes were incubated with calcein acetoxymethyl ester (calcein‐AM; green) and ethidium homodimer‐1 (EthD‐1; red) to stain live and dead cells respectively and imaged under fluorescent microscopy. Data are presented as averages ± standard deviation for *n* = 4 biological replicates with cells isolated from different animals. Significance is defined as *p* < 0.05 (*), *p* < 0.01 (**), and *p* < 0.001 (***).

Cytotoxicity of the polyP‐based particles was evaluated with primary bovine chondrocytes through a live‐dead assay, with the percentage of live and dead cells (**Figure** **7**) determined from fluorescence microscopy images (Figures S2 and S3, Supporting Information). As can be observed, the cytotoxicity of the polyP‐based particles was both concentration‐dependent and size‐dependent. While no significant differences in cell viability are observed for the larger, as synthesized particle agglomerates, the sonicated particles have appreciable cytotoxic effects at the highest concentration investigated of 4 ng cell^−1^, with significant differences compared to all other conditions. The untreated control had an average cell viability of 90.0 ± 3.3%, while chondrocytes treated with either sonicated Ca‐polyP and Sr‐polyP at 4 ng cell^−1^ exhibited a cell viability of 65.1 ± 10.0% and 70.9 ± 6.3%, respectively. It has been well established that smaller particles typically exhibit greater cytotoxic effects,^[^ ^58^, ^59^, ^60^ ^]^ with multiple studies reporting a decrease in cell viability for smaller particles and at higher particle concentrations.^[^ ^53^, ^61^, ^62^, ^63^, ^64^, ^65^, ^66^ ^]^ However, the level of cytotoxicity observed is not unexpected, given that the mass of a mammalian cell is estimated to be ≈3–4 ng,^[^ ^67^ ^]^ which is roughly equivalent to the mass of particles applied per cell at the highest concentration tested. These results suggest that the polyP‐based particles should be well tolerated for applications in drug delivery into the joint. Subsequent biological studies were performed under culture conditions in which minimal cytotoxicity would be expected, with particle concentrations of 0.125–0.5 ng cell^−1^. This was nonetheless verified with results indicating no significant differences in cell viability for Ca‐polyP and Sr‐polyP at all particle concentrations (Figures S4–S6, Supporting Information). Under these conditions, the average cell viability of the control was 96.6 ± 2.1%, while the average cell viability of all treated conditions combined (0.125–0.5 ng cell^−1^) was 95.4 ± 0.3%.

**Figure 8:**
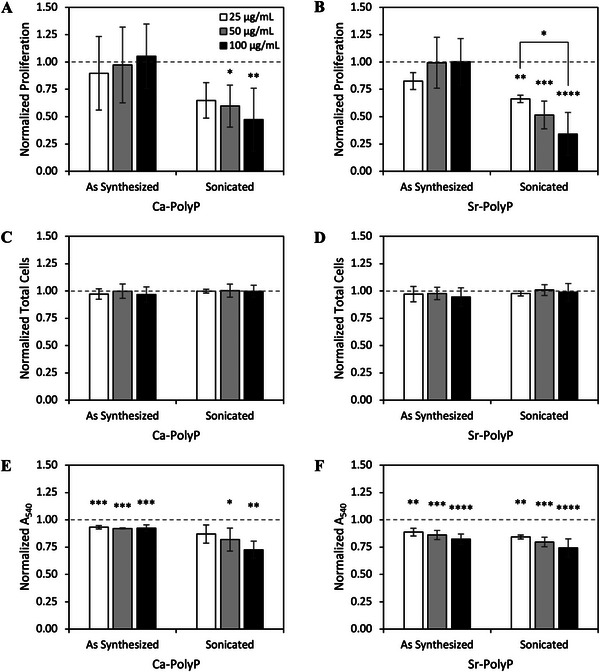
Bioactivity of polyP‐based particles. Primary bovine chondrocytes were treated with varying concentrations of Ca‐polyP and Sr‐polyP particles for 24 h, and A,B) cell proliferation, C,D) total number of cells, and E,F) metabolic activity were measured and normalized to the control samples cultured in the absence of particles, represented by the dotted lines. Data are presented as averages ± standard deviation for *n* = 4 biological replicates with cells isolated from different animals. Significance is defined as *p* < 0.05 (*), *p* < 0.01 (**), *p* < 0.001 (***), and *p* < 0.0001 (****).

We then investigated whether these polyP‐based particles could elicit certain bioactivities in chondrocytes. Previous work had demonstrated a reduction in proliferation for 3D chondrocyte cultures over time when treated with soluble polyP.^[^ ^13^ ^]^ The EdU cell proliferation assay was performed following treatment of chondrocytes with as synthesized or sonicated Ca‐polyP or Sr‐polyP particles and imaged by fluorescent microscopy to confirm this effect (Figures S7 and S8, Supporting Information). The percentage of proliferating cells was normalized to the untreated control samples (**Figure** **8**A,B). Despite the as synthesized particle agglomerates having little effect on cellular proliferation, the sonicated particles resulted in a significant decrease in cell proliferation at 50 and 100 µg mL^−1^ for Ca‐polyP and at all concentrations of Sr‐polyP compared to the control. Furthermore, there appears to be a concentration‐dependent effect, with a greater reduction in cell proliferation as the particle concentration is increased. However, this is only significant between 25 and 100 µg mL^−1^ of sonicated Sr‐polyP (*p* < 0.05). These results suggest that polyP maintains its ability to affect cell proliferation when presented in particle form. However, the fact that as synthesized particle agglomerates did not elicit this effect hints that the mechanism by which polyP inhibits chondrocyte proliferation has not been identified. Conversely, polyP increased cell proliferation in human and mouse fibroblast cells through the FGF signaling pathway,^[^ ^68^ ^]^ as well as in human breast cancer cell lines via the mTOR signaling pathway.^[^ ^69^ ^]^ As a result of these differences, it has been suggested that polyP has cell‐specific effects.^[^ ^13^ ^]^ Whole‐well fluorescent imaging was also performed to determine changes in cell numbers by counting total nuclei stained with Hoechst 33342 (Figure 8C,D), which indicated no differences in cell numbers between conditions following 1 day of culture with the particles. This was expected given that the culture conditions selected for these experiments result in limited chondrocyte proliferation to ensure that the process of chondrocyte dedifferentiation is slowed to reduce its impact on the results of the study.

MTT assays were performed on primary bovine chondrocytes following incubation with varying concentrations of the as synthesized or sonicated Ca‐polyP or Sr‐polyP particles to investigate a potential role in metabolism (Figure 8E,F). Statistically significant decreases in formazan production were observed in almost all conditions compared to the control, suggesting that these polyP‐based particles are bioactive and may reduce the metabolic activity per chondrocyte, as measured by this assay. This is further validated given that there were no differences in cytotoxic effects under these culture conditions (Figures S4–S6, Supporting Information) and no changes in cell numbers within the time frame of the experiment (Figure 8C,D). Interestingly, cellular polyP levels have been linked to mitochondrial activity, with decreased polyP levels as a result of mitochondrial inhibition, and increased polyP levels from supplementation of glutamine and pyruvate, both substrates of complex I, as well as supplementation of succinate, a substrate of complex II.^[^ ^70^ ^]^ F_0_F_1_ ATP synthase and ATPase activity have also been proposed to be involved in the synthesis and hydrolysis of polyP respectively.^[^ ^71^ ^]^ While the exact mechanism for this observed decrease in metabolic activity is not known, our results suggest that polyP modulates metabolic activity in chondrocytes. It is thought that this decrease in metabolic activity may be a compensatory response in chondrocytes by reducing endogenous polyP and ATP synthesis, given that the production of polyP has been linked to mitochondrial activity and that a source of bioenergetic molecules may be supplied in excess via the delivery of polyP‐based particles.

**Figure 9:**
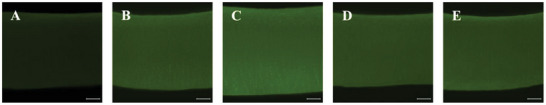
Cartilage tissue uptake of polyP‐based particles. Full‐thickness bovine cartilage explants were incubated with 100 µg mL^−1^ of DAPI‐stained particles (green) for 3 days at 37 °C. Representative fluorescent microscopy images of thin cross‐sections for tissues incubated A) in the absence of particles or with B) Ca‐polyP as synthesized or C) sonicated, and D) Sr‐polyP as synthesized or E) sonicated. Tissues are oriented with the superficial surface at the top. Scale bar = 100 µm.

To evaluate these polyP‐based particles further for delivery applications into cartilage tissue, full‐thickness cartilage explants were incubated in a bath containing DAPI‐stained particles according to protocols modified from others.^[^ ^72^, ^73^, ^74^, ^75^ ^]^ After 3 days of incubation, thin cross‐sections of the cartilage explants were imaged under fluorescent microscopy (**Figure** **9**). The fluorescent green signal is higher at all depths of cartilage for both types of the as synthesized and sonicated particles compared to explants that were incubated in the absence of the particles, suggesting successful uptake of the particles into the tissue. Increased fluorescence intensity was also noted in association with chondrocytes, suggesting preferential accumulation. As an additional validation, the fluorescent signal of the incubating bath was compared to an identical solution incubated in the absence of cartilage tissue to calculate the percentage of particle uptake, which indicated a drop in fluorescence of the incubating bath (Figure S9A, Supporting Information). Particle retention was also evaluated by incubating these cartilage explants in a fresh solution without particles. The fluorescent signal of the solution was then compared to the initial particle solution incubated in the absence of cartilage tissue to calculate particle retention within the tissue as a percentage of the amount initially uptaken (Figure S9B, Supporting Information), suggesting that a proportion of uptaken particles was retained within cartilage tissue for a period of at least 3 days. It is unclear at this point whether the relatively high tissue uptake levels measured for as synthesized particles involved the dispersion of agglomerates in a first event.

Due to the fixed negative charge of cartilage, the negative zeta potential of these polyP‐based particles was initially speculated to potentially pose as a barrier of entry into the tissue, as a result of electrostatic repulsion. Indeed, current research is focused extensively on the development of cationic drug carriers to facilitate penetration and retention within the tissue.^[^ ^7^ ^]^ Nevertheless, the effect of surface charge is complex given that intra‐articular injection of drug carriers first involves interactions with the synovial fluid. For instance, one study found that cationic particles aggregated in synovial fluid unlike anionic particles, and interestingly, aggregation was not solely due to hyaluronic acid.^[^ ^76^ ^]^ While particle uptake was improved for these cationic particles in healthy cartilage tissues, minimal differences between the uptake of cationic and anionic particles were observed in the OA model of cartilage, and this may be attributed to the reduced fixed charge density due to proteoglycan depletion.^[^ ^76^ ^]^ From our ex vivo study, it is suggested that these polyP‐based particles are capable of penetrating into and being retained within cartilage tissue explants despite exhibiting a negative zeta potential. As such, there can be a role of negatively charged drug carriers in the delivery of therapeutics to cartilage, and further studies into the effect of surface charge on the uptake, retention, and localization of nanoparticles are warranted. Furthermore, it is important to note that as the proposed application of these particles involves delivery to OA‐afflicted cartilage, it is thought that penetration may be improved as a result of ECM degeneration or tissue defects that may be present.^[^ ^10^ ^]^

## 3 Conclusion

In this study, Ca‐polyP and Sr‐polyP particles were synthesized by dropwise addition of Na‐polyP to an agitated divalent cation bath and characterized for feasibility as a drug delivery strategy in articular cartilage. This work presents an in‐depth physicochemical characterization of polyP‐based particles, as well as their size stability under a range of environmental conditions. Sonication successfully broke up particle agglomerates to a submicron range, and the particles exhibit a negative surface charge. Furthermore, the particles have a relatively spherical morphology. While these polyP‐based particles exhibit relatively poor size stability in a variety of media, with Ca‐polyP particles exhibiting lower stability than Sr‐polyP, this can be mitigated with FBS supplementation. These polyP‐based particles have also been demonstrated to exhibit biological effects in chondrocytes through the reduction of cell proliferation and metabolic activity, with cytotoxic effects observed only at high concentrations. Lastly, an ex vivo study also suggests successful penetration and retention in cartilage tissue explants, providing additional evidence for the uptake of negatively charged particles into the tissue. Taken together, these results provide a basis for the further development of polyP‐based particles for drug delivery into the joint.

## 4 Experimental Section

### Materials

Sodium polyphosphate (with an average chain length of 40 orthophosphate units) was a kind gift from Budenheim (Germany).

### PolyP‐Based Particle Synthesis

Ca‐polyP and Sr‐polyP particles were synthesized by dropwise addition of Na‐polyP into the corresponding cross‐linking solution, according to a protocol adapted from the works of Donovan et al.^[^ ^77^ ^]^ and Müller et al.^[^ ^30^ ^]^ Briefly, 5 mL of 4% w/v Na‐polyP at pH 10 was added at a rate of 60 mL h^−1^ using a syringe pump to 5 mL of 0.762 m CaCl_2_ or SrCl_2_ at pH 10. Concurrently, 2 mL of 1 m NaOH was added dropwise at a rate of 25 mL h^−1^ using a syringe pump to maintain the pH above 10 during synthesis. The solution was stirred continuously at 300 rpm for 3 h, and the particles were subsequently washed by centrifugation and resuspension in diH_2_O for a total of five washes and stored in 70% ethanol at −20 °C until further use.

### Particle Dispersion by Probe Sonication

Sonication was performed using a Q500 Sonicator (Qsonica) equipped with a microtip probe having a 1.6 mm tip diameter (#4417, Qsonica). The particles were diluted to an appropriate concentration in diH_2_O, and sonication was performed on ice at 50% amplitude for 30 s on and 30 s off, repeated for a total of ten cycles.

### Particle Size and Surface Charge Characterization

Particle size was measured in polystyrene cuvettes (Fisher Scientific) by DLS while zeta potential was measured in folded capillary zeta cells (Malvern Panalytical) with a Zetasizer Nano ZS (Malvern Panalytical) at a temperature of 25 °C. DLS measurements were performed daily for a period of 4 days in diH_2_O and varying NaCl concentrations while stored at room temperature. For the pH experiment, the particles were sonicated and stored in 8 mm Tris‐HCl at the corresponding pH. DLS measurements were also performed in low glucose (1 g L^−1^ glucose) DMEM (Corning) supplemented with 3.7 g L^−1^ NaHCO_3_, and 100 units mL^−1^ penicillin, 100 µg mL^−1^ streptomycin and 250 ng mL^−1^ amphotericin B (1X antibiotic‐antimycotic; Gibco), with or without 10% FBS (VWR) and incubated in a cell incubator maintained at 37 °C, 95% relative humidity and 5% CO_2_ over a 4 day period.

### SEM and Elemental Analysis

Particles were sonicated in diH_2_O and dried directly on aluminum stubs for imaging, while the as synthesized particles were dried on conductive tape for elemental analysis. Samples were sputter coated with gold using a Q150T ES (Quorum Technologies) at a thickness below 10 nm and imaged using a VEGA‐II XMU Scanning Electron Microscope (TESCAN) equipped with an INCA Energy X‐Act (Oxford Instruments) spectroscope for elemental analysis by EDX. Particle diameters were estimated from SEM images using ImageJ (NIH; version 1.51).

### FTIR Spectroscopy

FTIR was performed on dried particles using a Cary 630 FTIR Spectrophotometer with an attenuated total reflectance (ATR) sampling module (Agilent) at a resolution of 2 cm^−1^. The spectra of Ca‐polyP and Sr‐polyP were compared to the Na‐polyP starting material.

### Cartilage Tissue Extraction and Chondrocyte Isolation

Full‐thickness articular cartilage was extracted aseptically from the metacarpophalangeal joints of healthy 2‐ to 4‐year old cows sourced from a local abattoir (Tom Henderson Meats and Abattoir Inc.) within 24 h of death. For chondrocyte isolation, the excised cartilage was maintained in high glucose DMEM (4.5 g L^−1^ glucose) supplemented with 1X antibiotic‐antimycotic for 3 days. The cartilage tissue was then enzymatically digested, first with 0.2% w/v Pronase protease (EMD Millipore) in high glucose DMEM for 2 h, followed by three washes in DMEM. The cartilage tissue was subsequently digested with 0.1% w/v collagenase (Sigma‐Aldrich) in high glucose DMEM for 1 or 2 days in a cell incubator. The digest was passed through a 100 µm cell strainer, and the cells were washed three times in fresh DMEM by centrifugation and resuspension. Chondrocytes were then resuspended in low glucose DMEM supplemented with 3.7 g L^−1^ NaHCO_3_, 1X antibiotic‐antimycotic, and 10% heat‐inactivated FBS prior to cell seeding. For tissue penetration and retention experiments, the excised cartilage was punched into 6 mm diameter full‐thickness discs with biopsy punches (Integra Miltex). The tissues were briefly maintained in PBS (Sigma‐Aldrich) until further use.

### Determination of Cellular Particle Uptake

Ca‐polyP and Sr‐polyP particles were diluted to 2 mg mL^−1^ in diH_2_O and incubated with 0.1 mg mL^−1^ of DAPI overnight at 4 °C. Binding of DAPI to polyP shifts the fluorescence emission peak to the yellow‐green range of the spectrum, permitting the visualization of polyP distinctly from DNA and GAGs.^[^ ^78^ ^]^ DAPI‐stained particles were washed by centrifugation and resuspension five times in sterile diH_2_O, and then used as is or sonicated as described previously. Chondrocytes were seeded onto 24‐well tissue culture plates at 400 000 cells per well. Following 1 day, the medium was replaced with 2 mL of fresh medium containing 100 µg mL^−1^ of DAPI‐stained particles. After 1 day of treatment, the cells were washed three times with Hanks’ Balanced Salt Solution (HBSS; Wisent Bioproducts). Trypan blue was added to the HBSS at a final concentration of 0.1% w/v and incubated for 1 min prior to imaging to quench extracellular green fluorescence.^[^ ^79^, ^80^ ^]^ Cells were visualized under fluorescent microscopy using an Axio Observer 7 (Zeiss).

### Cell Viability Following Treatment with Particles

Chondrocytes were seeded onto 24‐well tissue culture plates at 25 000 cells per well. Following 1 day of cell attachment, the culture medium was replaced with 1 mL of fresh medium containing 0–100 µg mL^−1^ of Ca‐polyP or Sr‐polyP, corresponding to 0–4 ng cell^−1^. After 1 day of treatment, cells were stained with 2 µm of calcein‐AM (Life Technologies) and 4 µm of EthD‐1 (Sigma‐Aldrich) in culture medium and incubated at 37 °C for 30 min. Cells were visualized under fluorescent microscopy using the Axio Observer 7, and live and dead cells were counted using ImageJ from five images taken per condition at randomly selected locations. This was similarly repeated in 48‐well tissue culture plates at 80 000 cells per well using 400 µL of medium, corresponding to 0–0.5 ng cell^−1^ to match the culture conditions of other experiments to verify cytotoxicity under these conditions.

### Cell Responses Following Treatment with Particles

Chondrocytes were seeded onto 48‐well tissue culture plates at 80 000 cells per well for the cell proliferation assay, and under identical culture conditions in technical triplicates for the metabolic activity assay. Following 1 day of cell attachment, the culture medium was replaced with 400 µL of fresh medium containing 0–100 µg mL^−1^ of Ca‐polyP or Sr‐polyP.

To evaluate cell proliferation, after 1 day of treatment with the particles, 300 µL of medium in each well was removed and replaced with 100 µL of fresh medium without particles containing 20 µm of 5‐ethynyl‐2′‐deoxyuridine (EdU) in dimethyl sulfoxide (DMSO) and incubated for 4 h. Cells were fixed in 3.7% formaldehyde in PBS for 15 min and washed twice in 3% bovine serum albumin (BSA) in PBS. The cells were then permeabilized in 0.5% Triton X‐100 in PBS for 20 min on an orbital rocker, followed by two washes in 3% BSA in PBS. EdU detection was performed using the Click‐iT EdU Cell Proliferation Kit for Imaging (Invitrogen) as per the manufacturer's protocol using an Alexa Fluor 488 azide. The cells were washed once with 3% BSA in PBS, followed by a second wash using PBS. Nuclear staining was performed using 5 µg mL^−1^ of Hoechst 33342 in PBS for 30 min on an orbital rocker, protected from light. The cells were washed twice in PBS prior to imaging with the Axio Observer 7, and proliferating and total cells were counted using ImageJ from five images taken per condition at randomly selected locations. Total cell numbers were also quantified by imaging the entire surface of the well with the Axio Observer 7 and counting the total number of nuclei using ImageJ.

To evaluate metabolic activity, after 1 day of treatment with the particles, the medium was replaced with 100 µL of fresh medium without particles. Subsequently, 10 µL of 12 mm 3‐(4,5‐dimethylthiazol‐2‐yl)−2,5‐diphenyltetrazolium bromide (MTT; Calbiochem) in sterile PBS was added to each well. The cells were incubated for 4 h, after which 220 µL of DMSO was added and incubated for an additional 10 min at 37 °C. Each well was then thoroughly mixed and 100 µL was transferred to 96‐well plates in triplicates. Absorbance was read at 540 nm using a Synergy H1 Microplate Reader (BioTek).

### Evaluation of Cartilage Tissue Penetration and Retention

Cartilage discs were incubated in 400 µL of fresh PBS supplemented with 10% heat‐inactivated FBS and 100 µg mL^−1^ of DAPI‐stained particles for 3 days on an orbital rocker at 37 °C. This time point was selected based on the typical retention times of nanoparticles in the joint ranging from several days to weeks.^[^ ^81^ ^]^ The fluorescence of the solution was then measured at 415 nm excitation and 485 nm emission using a Synergy H1 Microplate Reader and the signal was compared to the corresponding solution incubated without cartilage tissue as the control. Similarly, PBS supplemented with 10% heat‐inactivated FBS incubated with and without the cartilage explants were used as respective blanks. Some of the cartilage explants were briefly rinsed in PBS and subsequently cut into thin slices using a custom‐made device comprising a number 22 scalpel blade and a cryotome blade, and tissue fluorescence was observed through its cross‐section using an Axio Observer 7. Meanwhile, all remaining explants were briefly rinsed in PBS, transferred to 400 µL of fresh PBS supplemented with 10% heat‐inactivated FBS, and incubated for an additional 3 days on an orbital rocker at 37 °C to assess retention of the particles within the tissue. Fluorescence of the solution was measured as previously described and compared to the initial fluorescent particle solution incubated without cartilage tissue.

### Statistical Analysis

Data are presented as means ± standard deviation. Statistical analyses were performed with SPSS (IBM; version 28.0.1.1). Pairwise comparisons were performed within groups using one‐way ANOVA with post‐hoc Tukey's HSD. The level of significance was set at *p* < 0.05 (*), *p* < 0.01 (**), *p* < 0.001 (***), and *p* < 0.0001 (****).

## Conflict of Interest

The authors declare no conflict of interest.

## Author Contributions

J.N. performed conceptualization, methodology, investigation, formal analysis, and wrote the original draft; N.S., K.V.S., and J.Y. performed investigation, and reviewed and edited the final manuscript; J.P.S. performed conceptualization, methodology, supervision, funding acquisition, and reviewed and edited the final manuscript.

## Supporting information

Supporting Information

## Data Availability

The data that support the findings of this study are available from the corresponding author upon reasonable request.

## References

[1] D. J. Hunter , S. Bierma‐Zeinstra , Lancet 2019, 393, 1745. 31034380 10.1016/S0140-6736(19)30417-9

[2] D. Primorac , V. Molnar , V. Matisic , D. Hudetz , Z. Jelec , E. Rod , F. Cukelj , D. Vidovic , T. Vrdoljak , B. Dobricic , D. Anticevic , M. Smolic , M. Miskulin , D. Cacic , I. Boric , Pharmaceuticals 2021, 14, 205. 33801304 10.3390/ph14030205 PMC8001498

[3] C. H. Evans , V. B. Kraus , L. A. Setton , Nat. Rev. Rheumatol. 2014, 10, 11. 24189839 10.1038/nrrheum.2013.159 PMC4402210

[4] I. A. Jones , R. Togashi , M. L. Wilson , N. Heckmann , C. T. Vangsness , Nat. Rev. Rheumatol. 2019, 15, 77. 30498258 10.1038/s41584-018-0123-4 PMC6390843

[5] C. Larsen , J. Østergaard , S. W. Larsen , H. Jensen , S. Jacobsen , C. Lindegaard , P. H. Andersen , J. Pharm. Sci. 2008, 97, 4622. 18306275 10.1002/jps.21346

[6] L. Han , A. J. Grodzinsky , C. Ortiz , Annu. Rev. Mater. Res. 2011, 41, 133. 22792042 10.1146/annurev-matsci-062910-100431 PMC3392687

[7] A. G. Bajpayee , A. J. Grodzinsky , Nat. Rev. Rheumatol. 2017, 13, 183. 28202920 10.1038/nrrheum.2016.210

[8] J. Mahendran , J.‐P. St‐Pierre , in Nanoengineering Materials for Biomedical Uses, Springer International Publishing, Cham, 2019, pp. 81.

[9] A. Vedadghavami , C. Zhang , A. G. Bajpayee , Nano Today 2020, 34, 100898. 32802145 10.1016/j.nantod.2020.100898 PMC7425807

[10] C. D. Didomenico , M. Lintz , L. J. Bonassar , Nat. Rev. Rheumatol. 2018, 14, 393. 29899547 10.1038/s41584-018-0033-5

[11] S. N. J. Moreno , R. Docampo , PLoS Pathog. 2013, 9, e1003230. 23658515 10.1371/journal.ppat.1003230 PMC3642070

[12] Y. Desfougères , A. Saiardi , C. Azevedo , Biochem. Soc. Trans. 2020, 48, 95. 32049314 10.1042/BST20190328 PMC7054745

[13] J.‐P. St‐Pierre , Q. Wang , S. Q. Li , R. M. Pilliar , R. A. Kandel , Tissue Eng. Part A 2012, 18, 1282. 22429075 10.1089/ten.TEA.2011.0356

[14] J.‐P. St‐Pierre , R. M. Pilliar , M. D. Grynpas , R. A. Kandel , Acta. Biomater. 2010, 6, 3302. 20188870 10.1016/j.actbio.2010.02.033

[15] J.‐P. St‐Pierre , L. Gan , J. Wang , R. M. Pilliar , M. D. Grynpas , R. A. Kandel , Acta. Biomater. 2012, 8, 1603. 22222151 10.1016/j.actbio.2011.12.022

[16] X. Wang , M. Ackermann , E. Tolba , M. Neufurth , F. Wurm , Q. Feng , S. Wang , H. Schröder , W. Müller , Eur. Cell Mater. 2016, 32, 271. 27905661 10.22203/eCM.v032a18

[17] W. E. G. Müller , M. Ackermann , E. Tolba , M. Neufurth , S. Wang , H. C. Schröder , X. Wang , RSC Adv. 2016, 6, 88559. 10.22203/eCM.v032a18 27905661

[18] R. Gawri , R. Bielecki , E. W. Salter , A. Zelinka , T. Shiba , G. Collingridge , A. Nagy , R. A. Kandel , J. Orthop. Res. 2022, 40, 310. 33719091 10.1002/jor.25032

[19] P. Hu , J. Du , S. Zhang , T. Wang , J. Li , G. Chen , G. Zhou , Biol. Trace Elem. Res. 2020, 193, 422. 31054068 10.1007/s12011-019-01711-9

[20] J.‐P. Pelletier , M. Kapoor , H. Fahmi , D. Lajeunesse , A. Blesius , J. Maillet , J. Martel‐Pelletier , Ann. Rheum. Dis. 2013, 72, 250. 23065732 10.1136/annrheumdis-2012-201710

[21] J.‐P. Pelletier , C. Roubille , J.‐P. Raynauld , F. Abram , M. Dorais , P. Delorme , J. Martel‐Pelletier , Ann. Rheum. Dis. 2015, 74, 422. 24297379 10.1136/annrheumdis-2013-203989

[22] T. Jiang , H.‐M. Kan , K. Rajpura , E. J. Carbone , Y. Li , K. W.‐H. Lo , J. Nanosci. Nanotechnol. 2018, 18, 2310. 29442897 10.1166/jnn.2018.14311

[23] H. Huang , Z. Lou , S. Zheng , J. Wu , Q. Yao , R. Chen , L. Kou , D. Chen , Drug Deliv. 2022, 29, 767. 35261301 10.1080/10717544.2022.2048130 PMC8920370

[24] Y. Takechi‐Haraya , T. Ohgita , Y. Demizu , H. Saito , K.‐I. Izutsu , K. Sakai‐Kato , AAPS PharmSciTech 2022, 23, 150. 35596094 10.1208/s12249-022-02303-y PMC9122548

[25] S. Mahmood , U. K. Mandal , B. Chatterjee , M. Taher , Nanotechnol. Rev. 2017, 6, 355.

[26] N. Jurga , D. Przybylska , P. Kaminski , T. Grzyb , Sci. Rep. 2021, 11, 18846. 34552158 10.1038/s41598-021-98240-0 PMC8458358

[27] M.‐D. Yan , Y.‐J. Ou , Y.‐J. Lin , R.‐M. Liu , Y. Fang , W.‐L. Wu , L. Zhou , X. Yao , J. Chen , BMC Oral Health 2022, 22, 62. 35260122 10.1186/s12903-022-02092-7 PMC8905839

[28] K. Qiu , X. J. Zhao , C. X. Wan , C. S. Zhao , Y. W. Chen , Biomaterials 2006, 27, 1277. 16143392 10.1016/j.biomaterials.2005.08.006

[29] H. T. Phan , A. J. Haes , J. Phys. Chem. C 2019, 123, 16495. 10.1021/acs.jpcc.9b00913 PMC6913534 31844485

[30] W. E. G. Müller , E. Tolba , H. C. Schröder , S. Wang , G. Glaßer , R. Muñoz‐Espí , T. Link , X. Wang , Mater. Lett. 2015, 148, 163.

[31] W. E. G. Müller , E. Tolba , M. Ackermann , M. Neufurth , S. Wang , Q. Feng , H. C. Schröder , X. Wang , Acta Biomater. 2017, 50, 89. 28017868 10.1016/j.actbio.2016.12.045

[32] J. J. Christ , S. Willbold , L. M. Blank , Anal. Chem. 2020, 92, 4167. 32039586 10.1021/acs.analchem.9b05144

[33] R. A. French , A. R. Jacobson , B. Kim , S. L. Isley , R. L. Penn , P. C. Baveye , Environ. Sci. Technol. 2009, 43, 1354. 19350903 10.1021/es802628n

[34] I. P. D. Picola , K. A. N. Busson , A. H. Casé , F. D. Nasário , V. A. D. O. Tiera , S. R. Taboga , J. R. Neto , M. J. Tiera , J. Exp. Nanosci. 2013, 8, 703.

[35] L. Wang , X. Yang , Q. Wang , Y. Zeng , L. Ding , W. Jiang , J. Environ. Sci. 2017, 51, 248. 10.1016/j.jes.2016.07.003 28115136

[36] D. Bastos , F. J. De Las Nieves , Colloid Polym. Sci. 1993, 271, 860.

[37] M. Elimelech , C. R. O'melia , Colloids Surf. 1990, 44, 165.

[38] R. Folkersma , A. J. G. Van Diemen , H. N. Stein , Langmuir 1998, 14, 5973.

[39] P. Leroy , C. Tournassat , O. Bernard , N. Devau , M. Azaroual , J. Colloid Interface Sci. 2015, 451, 21. 25875489 10.1016/j.jcis.2015.03.047

[40] F. Yang , W. Wu , S. Chen , W. Gan , Soft Matter 2017, 13, 638. 27991633 10.1039/c6sm02174c

[41] R. Rampado , S. Crotti , P. Caliceti , S. Pucciarelli , M. Agostini , Front. Bioeng. Biotechnol. 2020, 8. 10.3389/fbioe.2020.00166 PMC7145938 32309278

[42] N. Singh , C. Marets , J. Boudon , N. Millot , L. Saviot , L. Maurizi , Nanoscale Adv. 2021, 3, 1209. 36132858 10.1039/d0na00863j PMC9416870

[43] A. Tomak , S. Cesmeli , B. D. Hanoglu , D. Winkler , C. Oksel Karakus , Nanotoxicology 2021, 15, 1331. 35061957 10.1080/17435390.2022.2025467

[44] P. Bihari , M. Vippola , S. Schultes , M. Praetner , A. G. Khandoga , C. A. Reichel , C. Coester , T. Tuomi , M. Rehberg , F. Krombach , Part. Fibre Toxicol. 2008, 5, 14. 18990217 10.1186/1743-8977-5-14 PMC2584664

[45] R. C. Murdock , L. Braydich‐Stolle , A. M. Schrand , J. J. Schlager , S. M. Hussain , Toxicol. Sci. 2008, 101, 239. 17872897 10.1093/toxsci/kfm240

[46] D. Mahl , C. Greulich , W. Meyer‐Zaika , M. Köller , M. Epple , J. Mater. Chem. 2010, 20, 6176.

[47] Z. Ji , X. Jin , S. George , T. Xia , H. Meng , X. Wang , E. Suarez , H. Zhang , E. M. V. Hoek , H. Godwin , A. E. Nel , J. I. Zink , Environ. Sci. Technol. 2010, 44, 7309. 20536146 10.1021/es100417s PMC3971839

[48] S. Kittler , C. Greulich , J. S. Gebauer , J. Diendorf , L. Treuel , L. Ruiz , J. M. Gonzalez‐Calbet , M. Vallet‐Regi , R. Zellner , M. Köller , M. Epple , J. Mater. Chem. 2010, 20, 512.

[49] H. T. R. Wiogo , M. Lim , V. Bulmus , L. Gutiérrez , R. C. Woodward , R. Amal , Langmuir 2012, 28, 4346. 22313424 10.1021/la204740t

[50] C. Graf , Q. Gao , I. Schütz , C. N. Noufele , W. Ruan , U. Posselt , E. Korotianskiy , D. Nordmeyer , F. Rancan , S. Hadam , A. Vogt , J. Lademann , V. Haucke , E. Rühl , Langmuir 2012, 28, 7598. 22524440 10.1021/la204913t

[51] T. L. Moore , L. Rodriguez‐Lorenzo , V. Hirsch , S. Balog , D. Urban , C. Jud , B. Rothen‐Rutishauser , M. Lattuada , A. Petri‐Fink , Chem. Soc. Rev. 2015, 44, 6287. 26056687 10.1039/c4cs00487f

[52] A. C. Sabuncu , J. Grubbs , S. Qian , T. M. Abdel‐Fattah , M. W. Stacey , A. Beskok , Colloids Surf B Biointerfaces 2012, 95, 96. 22421416 10.1016/j.colsurfb.2012.02.022

[53] Y. Ma , Y. Guo , S. Wu , Z. Lv , Q. Zhang , Y. Ke , RSC Adv. 2017, 7, 23560.

[54] M. P. Monopoli , D. Walczyk , A. Campbell , G. Elia , I. Lynch , F. Baldelli Bombelli , K. A. Dawson , J. Am. Chem. Soc. 2011, 133, 2525. 21288025 10.1021/ja107583h

[55] M. Schäffler , M. Semmler‐Behnke , H. Sarioglu , S. Takenaka , A. Wenk , C. Schleh , S. M. Hauck , B. D. Johnston , W. G. Kreyling , Nanotechnology 2013, 24, 265103. 23735821 10.1088/0957-4484/24/26/265103

[56] K. Partikel , R. Korte , D. Mulac , H.‐U. Humpf , K. Langer , Beilstein J. Nanotechnol. 2019, 10, 1002. 31165027 10.3762/bjnano.10.101 PMC6541368

[57] W. E. G. Müller , S. Wang , E. Tolba , M. Neufurth , M. Ackermann , R. Muñoz‐Espí , I. Lieberwirth , G. Glasser , H. C. Schröder , X. Wang , Small 2018, 14, 1801170. 10.1002/smll.201801170 29847707

[58] M. Akter , M. T. Sikder , M. M. Rahman , A. K. M. A. Ullah , K. F. B. Hossain , S. Banik , T. Hosokawa , T. Saito , M. Kurasaki , J Adv Res 2018, 9, 1. 30046482 10.1016/j.jare.2017.10.008 PMC6057238

[59] X. Dong , Z. Wu , X. Li , L. Xiao , M. Yang , Y. Li , J. Duan , Z. Sun , Int J Nanomedicine 2020, 15, 9089. 33244229 10.2147/IJN.S276105 PMC7683827

[60] H. I. Chiu , N. A. Samad , L. Fang , V. Lim , RSC Adv. 2021, 11, 9433. 35423427 10.1039/d1ra00074h PMC8695459

[61] M. V. D. Z. Park , A. M. Neigh , J. P. Vermeulen , L. J. J. De La Fonteyne , H. W. Verharen , J. J. Briedé , H. Van Loveren , W. H. De Jong , Biomaterials 2011, 32, 9810. 21944826 10.1016/j.biomaterials.2011.08.085

[62] A. R. Gliga , S. Skoglund , I. Odnevall Wallinder , B. Fadeel , H. L. Karlsson , Part. Fibre Toxicol. 2014, 11, 11. 24529161 10.1186/1743-8977-11-11 PMC3933429

[63] A. Ivask , I. Kurvet , K. Kasemets , I. Blinova , V. Aruoja , S. Suppi , H. Vija , A. Käkinen , T. Titma , M. Heinlaan , M. Visnapuu , D. Koller , V. Kisand , A. Kahru , PLoS One 2014, 9, e102108. 25048192 10.1371/journal.pone.0102108 PMC4105572

[64] D. Sahu , G. M. Kannan , M. Tailang , R. Vijayaraghavan , J. Nanosci. 2016, 2016, 4023852.

[65] Q. Feng , Y. Liu , J. Huang , K. Chen , J. Huang , K. Xiao , Sci. Rep. 2018, 8, 2082. 29391477 10.1038/s41598-018-19628-z PMC5794763

[66] Q. Xia , J. Huang , Q. Feng , X. Chen , X. Liu , X. Li , T. Zhang , S. Xiao , H. Li , Z. Zhong , K. Xiao , Int J Nanomed. 2019, 14, 6957. 10.2147/IJN.S214008 PMC6717860 32021157

[67] C. E. Sims , N. L. Allbritton , Lab Chip 2007, 7, 423. 17389958 10.1039/b615235j

[68] T. Shiba , D. Nishimura , Y. Kawazoe , Y. Onodera , K. Tsutsumi , R. Nakamura , M. Ohshiro , J. Biol. Chem. 2003, 278, 26788. 12740373 10.1074/jbc.M303468200

[69] L. Wang , C. D. Fraley , J. Faridi , A. Kornberg , R. A. Roth , Proc. Natl. Acad. Sci. USA 2003, 100, 11249. 12970465 10.1073/pnas.1534805100 PMC208743

[70] E. Pavlov , R. Aschar‐Sobbi , M. Campanella , R. J. Turner , M. R. Gómez‐García , A. Y. Abramov , J. Biol. Chem. 2010, 285, 9420. 20124409 10.1074/jbc.M109.013011 PMC2843191

[71] A. Y. Baev , P. R. Angelova , A. Y. Abramov , Biochem. J. 2020, 477, 1515. 32270854 10.1042/BCJ20200042 PMC7200627

[72] Y. Krishnan , H. A. Rees , C. P. Rossitto , S.‐E. Kim , H.‐H. K. Hung , E. H. Frank , B. D. Olsen , D. R. Liu , P. T. Hammond , A. J. Grodzinsky , Biomaterials 2018, 183, 218. 30173104 10.1016/j.biomaterials.2018.08.050 PMC6141342

[73] Y. Wei , L. Yan , L. Luo , T. Gui , B. Jang , A. Amirshaghaghi , T. You , A. Tsourkas , L. Qin , Z. Cheng , Sci. Adv. 2021, 7, eabe6374 33827816 10.1126/sciadv.abe6374 PMC8026133

[74] U. von Mentzer , T. Selldén , L. Råberg , G. Erensoy , A.‐K. Hultgård Ekwall , A. Stubelius 2022, 30, 1356. 10.1016/j.joca.2022.07.002 35840018

[75] J. Gong , J. Nhan , J.‐P. St‐Pierre , E. R. Gillies , J. Mater. Chem. B. 2023, 11, 8804. 37668597 10.1039/d3tb01417g

[76] S. Brown , J. Pistiner , I. M. Adjei , B. Sharma , Mol. Pharm. 2019, 16, 469. 28669194 10.1021/acs.molpharmaceut.7b00484 PMC7147813

[77] A. J. Donovan , J. Kalkowski , S. A. Smith , J. H. Morrissey , Y. Liu , Biomacromolecules 2014, 15, 3976. 25268994 10.1021/bm501046t PMC8808366

[78] R. A. Allan , J. J. Miller , Can. J. Microbiol. 1980, 26, 912. 7006768 10.1139/m80-158

[79] S. Sahlin , J. Hed , I. Runfquist , J. Immunol. Methods 1983, 60, 115. 6406600 10.1016/0022-1759(83)90340-x

[80] S. Vranic , N. Boggetto , V. Contremoulins , S. Mornet , N. Reinhardt , F. Marano , A. Baeza‐Squiban , S. Boland , Part. Fibre Toxicol. 2013, 10, 2. 23388071 10.1186/1743-8977-10-2 PMC3599262

[81] S. Brown , S. Kumar , B. Sharma , Acta. Biomater. 2019, 93, 239. 30862551 10.1016/j.actbio.2019.03.010 PMC6615949

