# Supplementary material for: Characterization of Calcium‐ and Strontium‐Polyphosphate Particles Toward Drug Delivery into Articular Cartilage
Source: Macromol Biosci. 2023 Oct 18;24(3):2300345. doi: 10.1002/mabi.202300345 (PMC13420767; doi:10.1002/mabi.202300345)
Supplement: Supplementary file 1 — Supporting Information [file MABI-24-2300345-s001.pdf]

Macromolecular Bioscience

Supporting Information

for *Macromol. Biosci.*, DOI 10.1002/mabi.202300345

Characterization of Calcium- and Strontium-Polyphosphate Particles Toward Drug Delivery into Articular Cartilage

*Jordan Nhan, Nicolas Strebel, Khushnouma Virah Sawmy, Jordan Yin and Jean-Philippe St-Pierre**

Supporting Information

Characterization of Calcium- and Strontium-Polyphosphate Particles Toward Drug Delivery into Articular Cartilage

*Jordan Nhan, Nicolas Strebel, Khushnouma Virah Sawmy, Jordan Yin, Jean-Philippe St-Pierre**

Author Affiliations
Department of Chemical and Biological Engineering, Faculty of Engineering, University of Ottawa, 161 Louis-Pasteur Pvt., Ottawa, Ontario, K1N 6N5, Canada

***Corresponding Author**
Jean-Philippe St-Pierre
Email: Jean-Philippe.St-Pierre@uOttawa.ca

Table S1. Estimated degree of ionization of the phosphate groups within polyphosphate.

**P-Subunit**	pH = 7.0	pH = 8.5	pH = 10.0	pH = 11.5
pK_a = 0.0	1.00	1.00	1.00	1.00
pK_a = 1.5	1.00	1.00	1.00	1.00
pK_a = 3.0	1.00	1.00	1.00	1.00
**End-Group**	pH = 7.0	pH = 8.5	pH = 10.0	pH = 11.5
pK_a = 7.0	0.50	0.97	1.00	1.00
pK_a = 8.0	0.09	0.76	0.99	1.00
pK_a = 9.0	0.01	0.24	0.91	1.00

Degree of ionization is defined as $[A^-]/[HA]$ and was estimated from the Henderson-Hasselbalch equation assuming each group behaves as a weak acid, using an estimated pK_a range for mid-chain phosphates (P-subunits) and end-groups on the polymer chain.

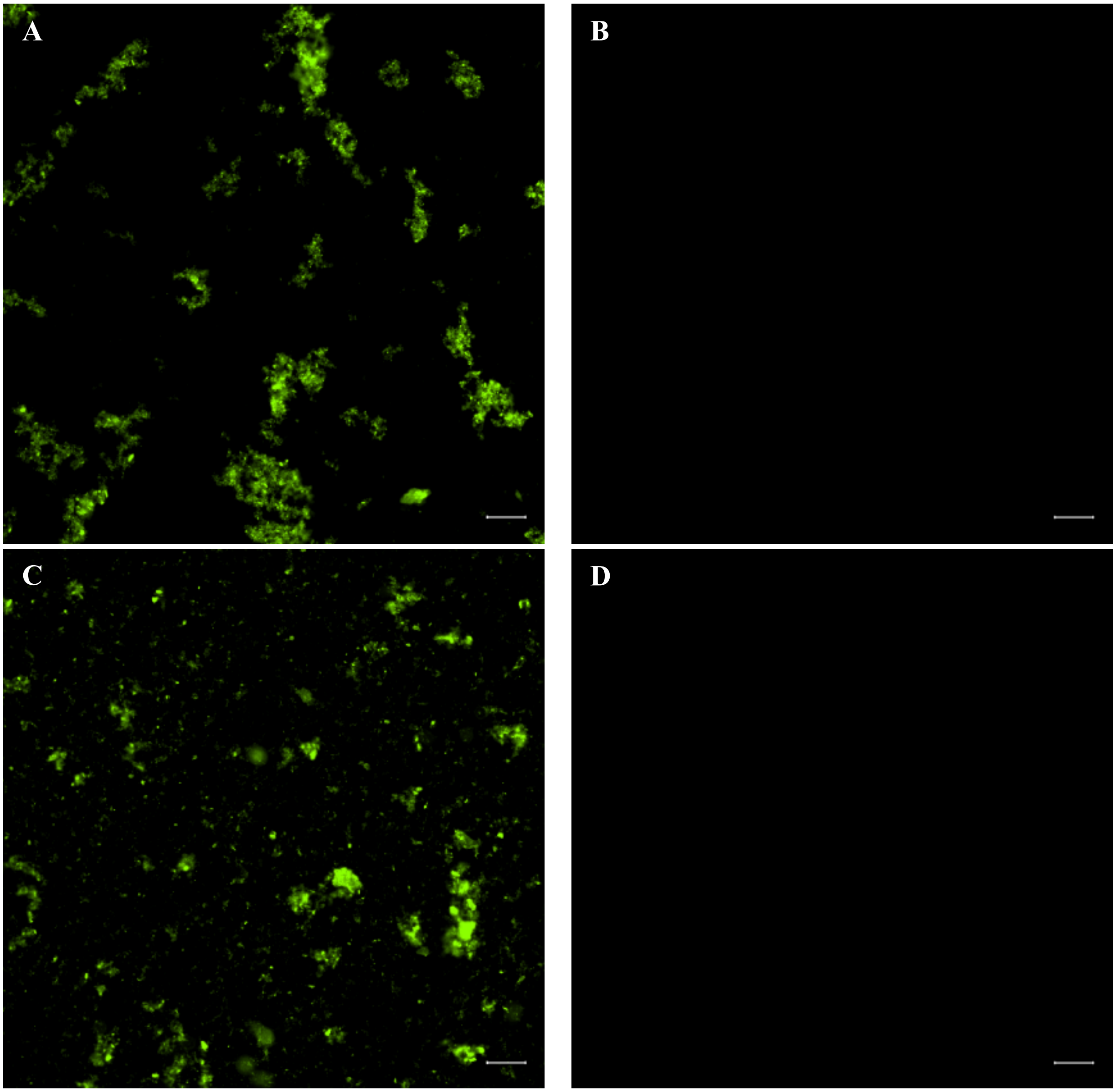

Figure S1. Trypan blue quenching of DAPI-stained polyP-based particles. DAPI-stained (green), as synthesized A) Ca-polyP and B) Ca-polyP incubated with 0.1% trypan blue along with C) Sr-polyP and D) Sr-polyP incubated with 0.1% trypan blue were imaged under fluorescent microscopy. Scale bar = 100 μm.

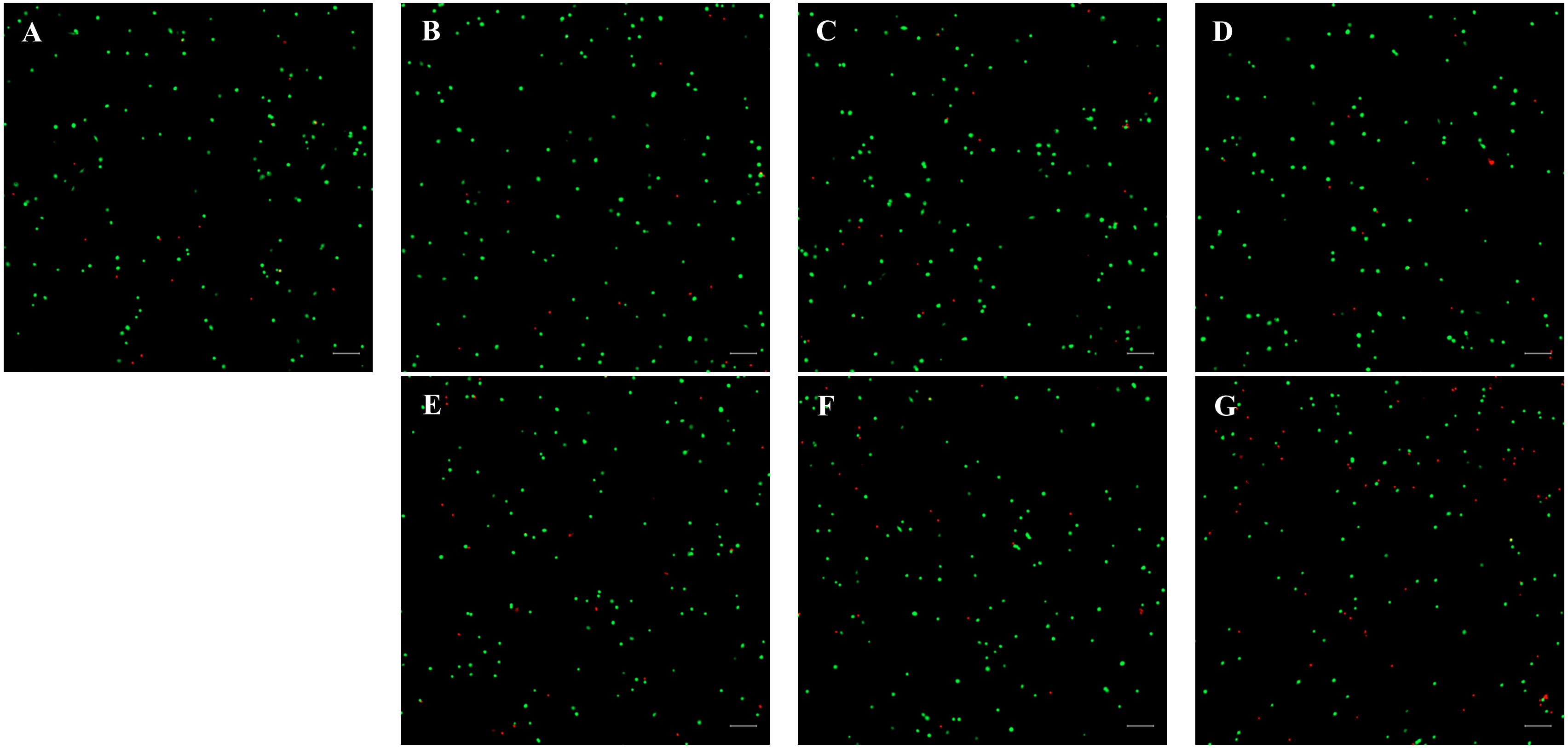

Figure S2. Representative images of live-dead assays on chondrocytes treated with Ca-polyP particles. Primary bovine chondrocytes were treated with A) no particles as the control, Ca-polyP as synthesized at B) 1 ng/cell, C) 2 ng/cell, and D) 4 ng/cell respectively, or sonicated Ca-polyP at a concentration of E) 1 ng/cell, F) 2 ng/cell, and G) 4 ng/cell respectively for 24 hours. Chondrocytes were incubated with both calcein-AM and EthD-1 to stain live (green) and dead (red) cells respectively. Scale bar = 100 μm.

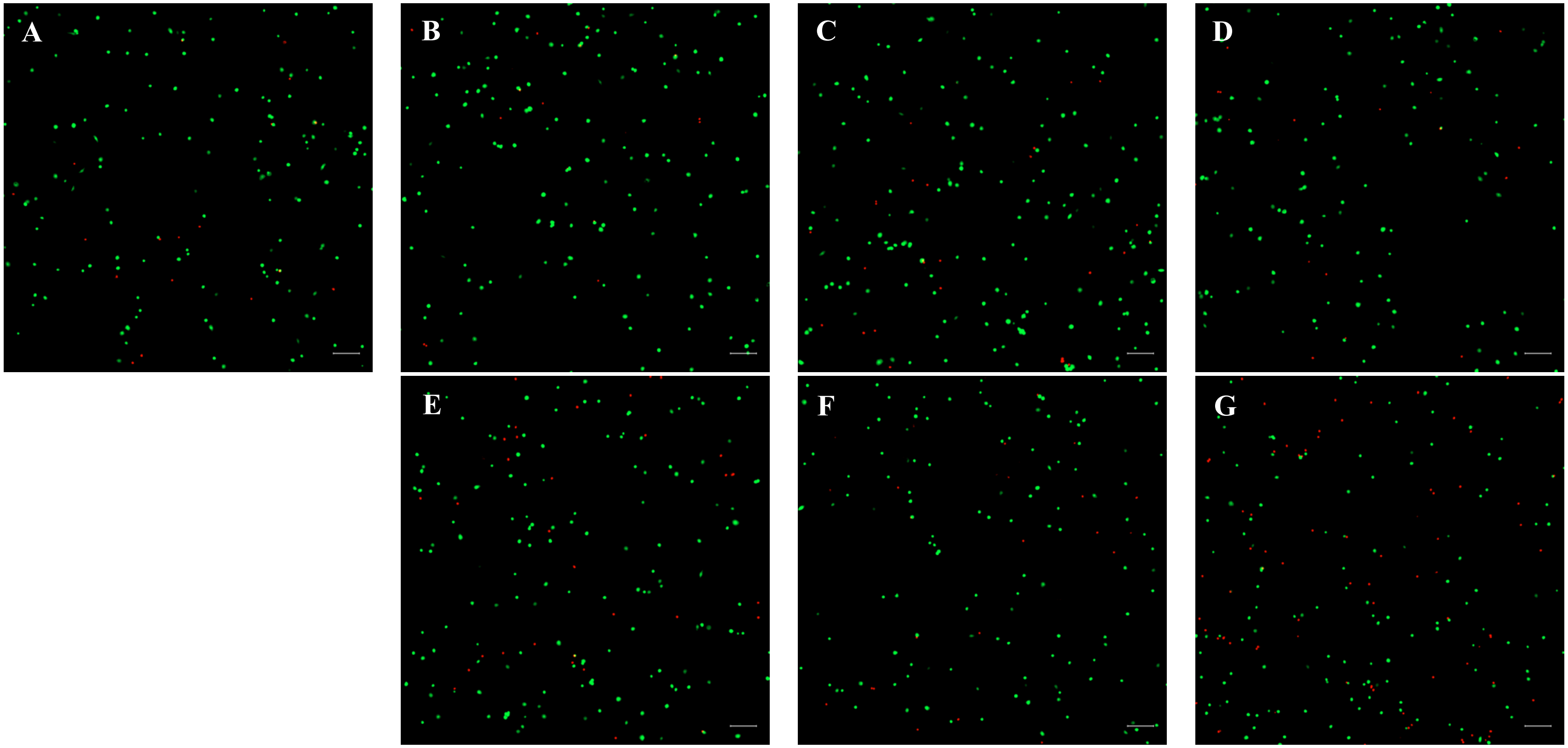

Figure S3. Representative images of live-dead assays on chondrocytes treated with Sr-polyP particles. Primary bovine chondrocytes were treated with A) no particles as the control, Sr-polyP as synthesized at B) 1 ng/cell, C) 2 ng/cell, and D) 4 ng/cell respectively, or sonicated Sr-polyP at a concentration of E) 1 ng/cell, F) 2 ng/cell, and G) 4 ng/cell respectively for 24 hours. Chondrocytes were incubated with both calcein-AM and EthD-1 to stain live (green) and dead (red) cells respectively. Scale bar = 100 μm.

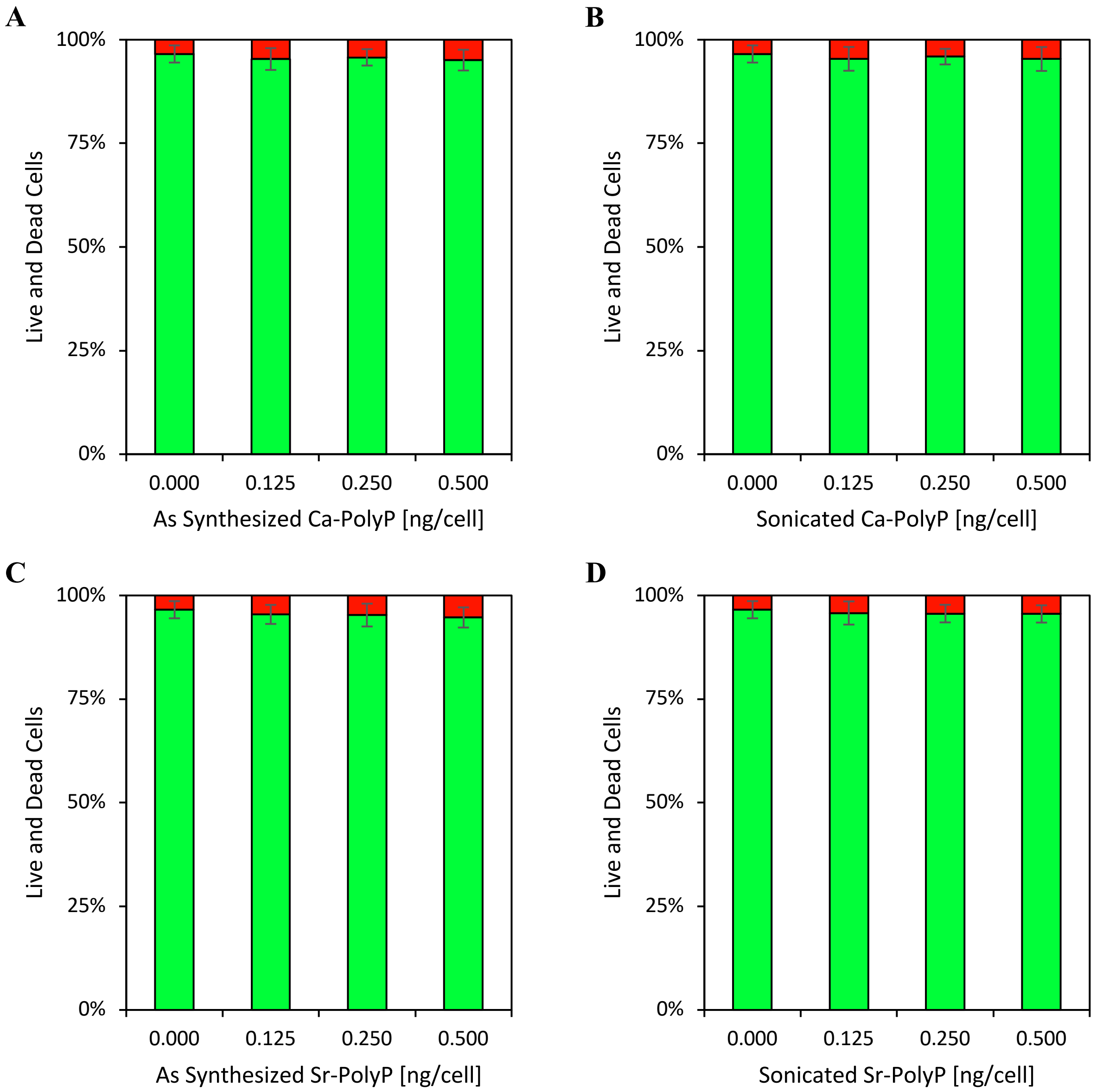

Figure S4. Live-dead assay under identical culture conditions of EdU and MTT assays. Primary bovine chondrocytes were treated with A) Ca-polyP as synthesized or B) sonicated and C) Sr-polyP as synthesized or D) sonicated at varying concentrations for 24 hours. Chondrocytes were incubated with calcein-AM (green) and EthD-1 (red) to stain live and dead cells respectively and imaged under fluorescent microscopy. Data are presented as averages ± standard deviation for n = 4 biological replicates with cells isolated from different animals.

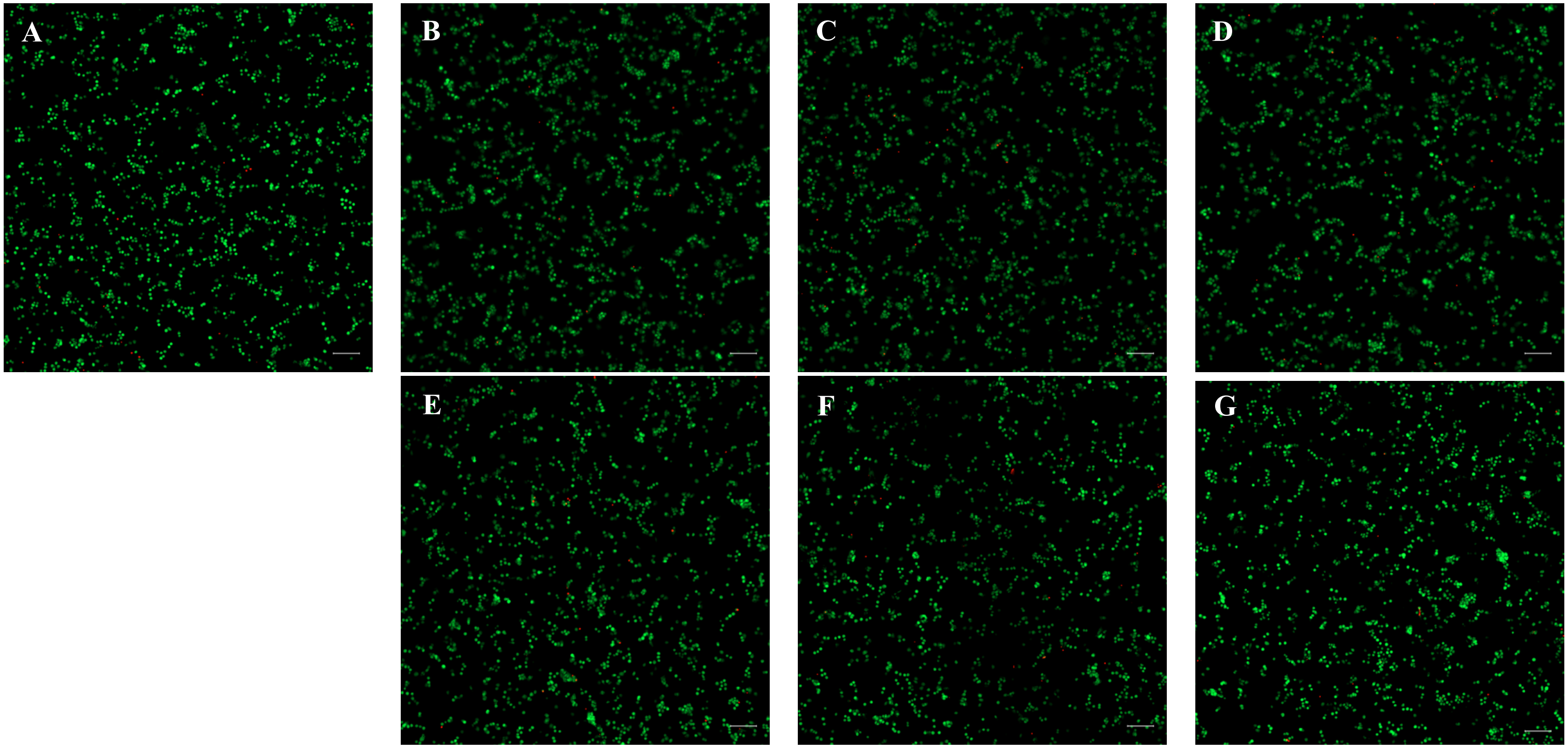

Figure S5. Representative images of live-dead assays on chondrocytes treated with Ca-polyP under identical culture conditions of EdU and MTT assays. Primary bovine chondrocytes were treated with A) no particles as the control, Ca-polyP as synthesized at B) 0.125 ng/cell, C) 0.25 ng/cell, and D) 0.5 ng/cell respectively, or sonicated Ca-polyP at a concentration of E) 0.125 ng/cell, F) 0.25 ng/cell, and G) 0.5 ng/cell respectively for 24 hours. Chondrocytes were incubated with both calcein-AM and EthD-1 to stain live (green) and dead (red) cells respectively. Scale bar = 100 μm.

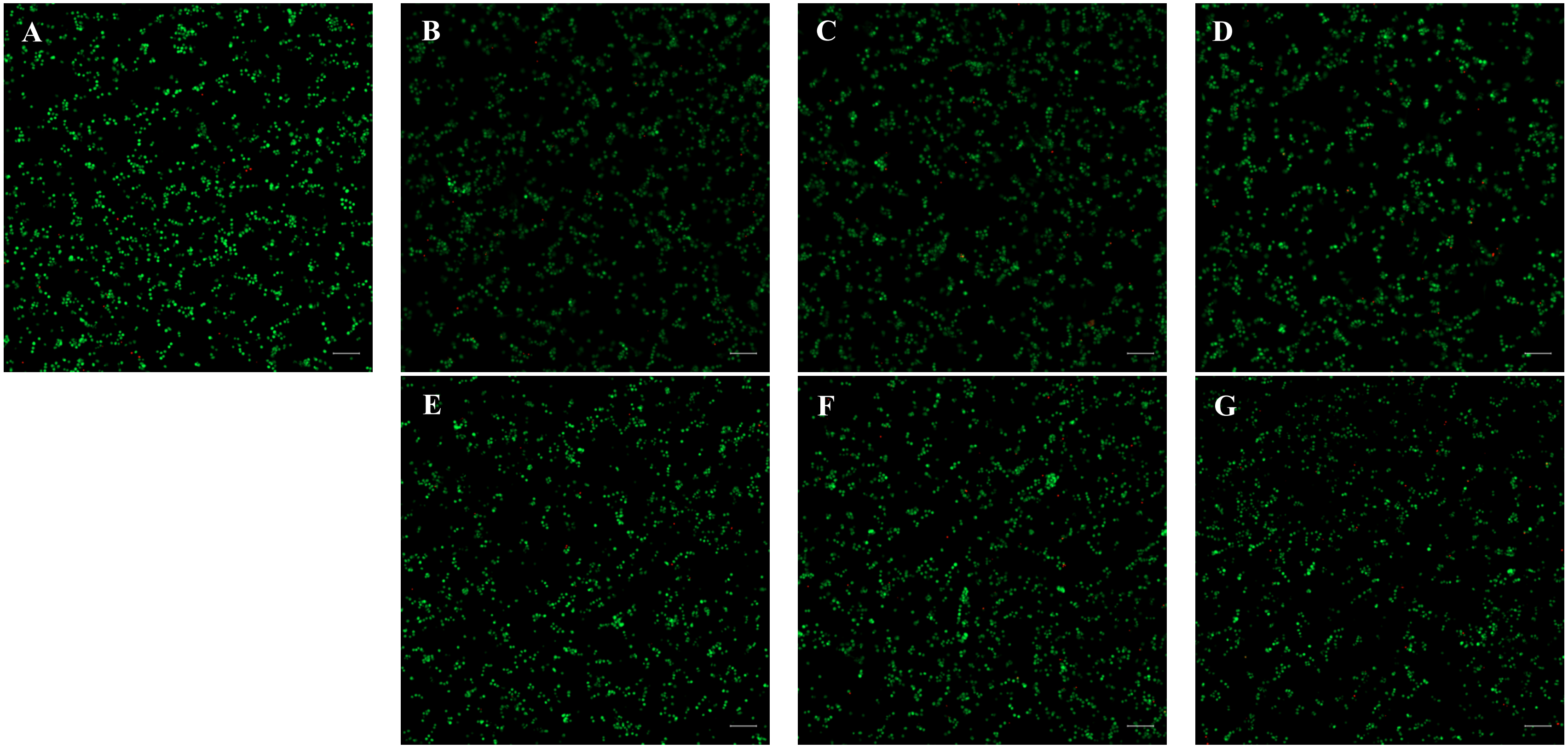

Figure S6. Representative images of live-dead assays on chondrocytes treated with Sr-polyP under identical culture conditions of EdU and MTT assays. Primary bovine chondrocytes were treated with A) no particles as the control, Sr-polyP as synthesized at B) 0.125 ng/cell, C) 0.25 ng/cell, and D) 0.5 ng/cell respectively, or sonicated Sr-polyP at a concentration of E) 0.125 ng/cell, F) 0.25 ng/cell, and G) 0.5 ng/cell respectively for 24 hours. Chondrocytes were incubated with both calcein-AM and EthD-1 to stain live (green) and dead (red) cells respectively. Scale bar = 100 μm.

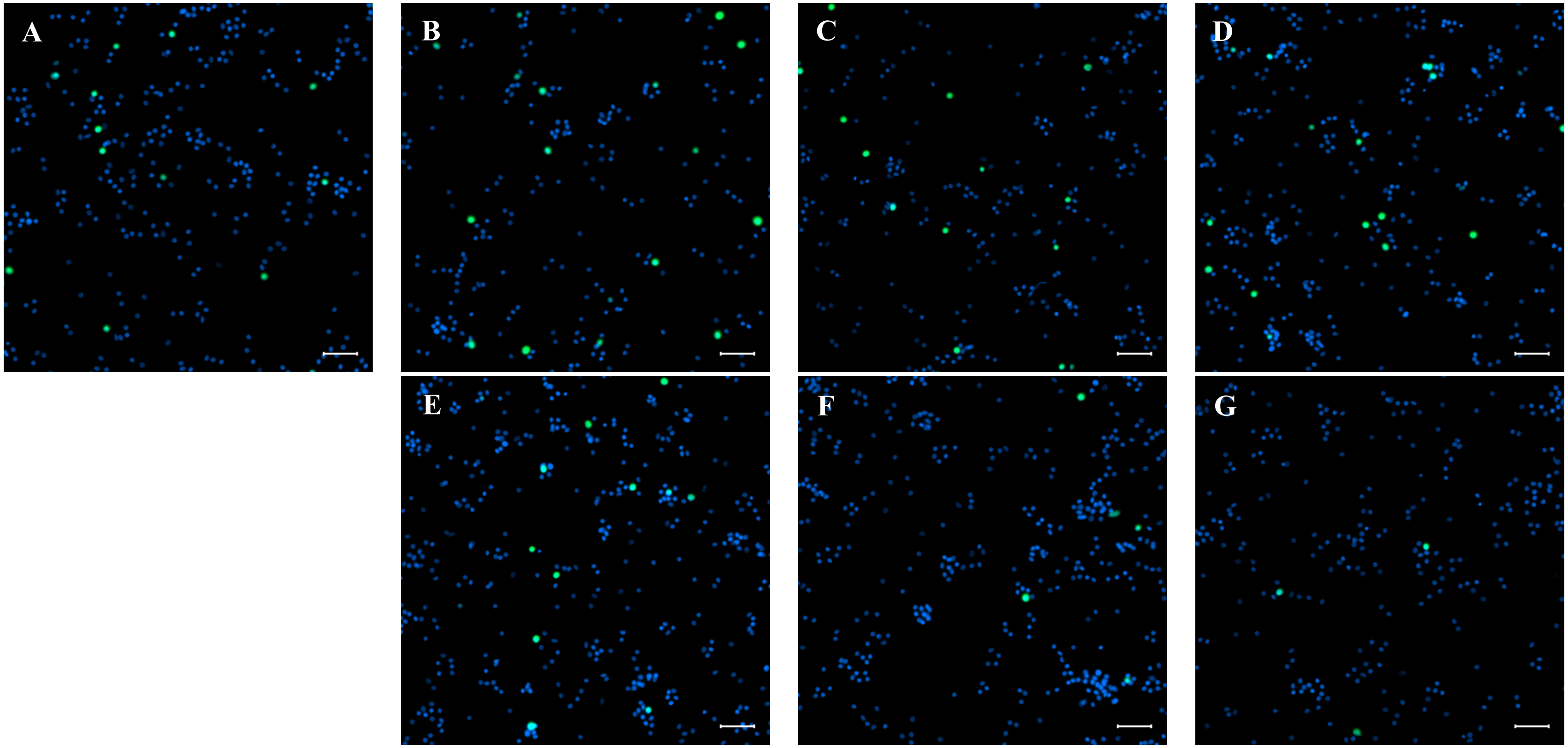

Figure S7. Representative images of EdU proliferation assays on chondrocytes treated with Ca-polyP. Primary bovine chondrocytes were treated with A) no particles as the control, Ca-polyP as synthesized at a concentration of B) 25 μg mL^{-1}, C) 50 μg mL^{-1}, and D) 100 μg mL^{-1} respectively, or sonicated Ca-polyP at a concentration of E) 25 μg mL^{-1}, F) 50 μg mL^{-1}, and G) 100 μg mL^{-1} respectively for 24 hours. Proliferating cells were labelled with an Alexa Fluor 488 azide (green) and nuclei were stained with Hoechst 33342 (blue). Scale bar = 50 μm.

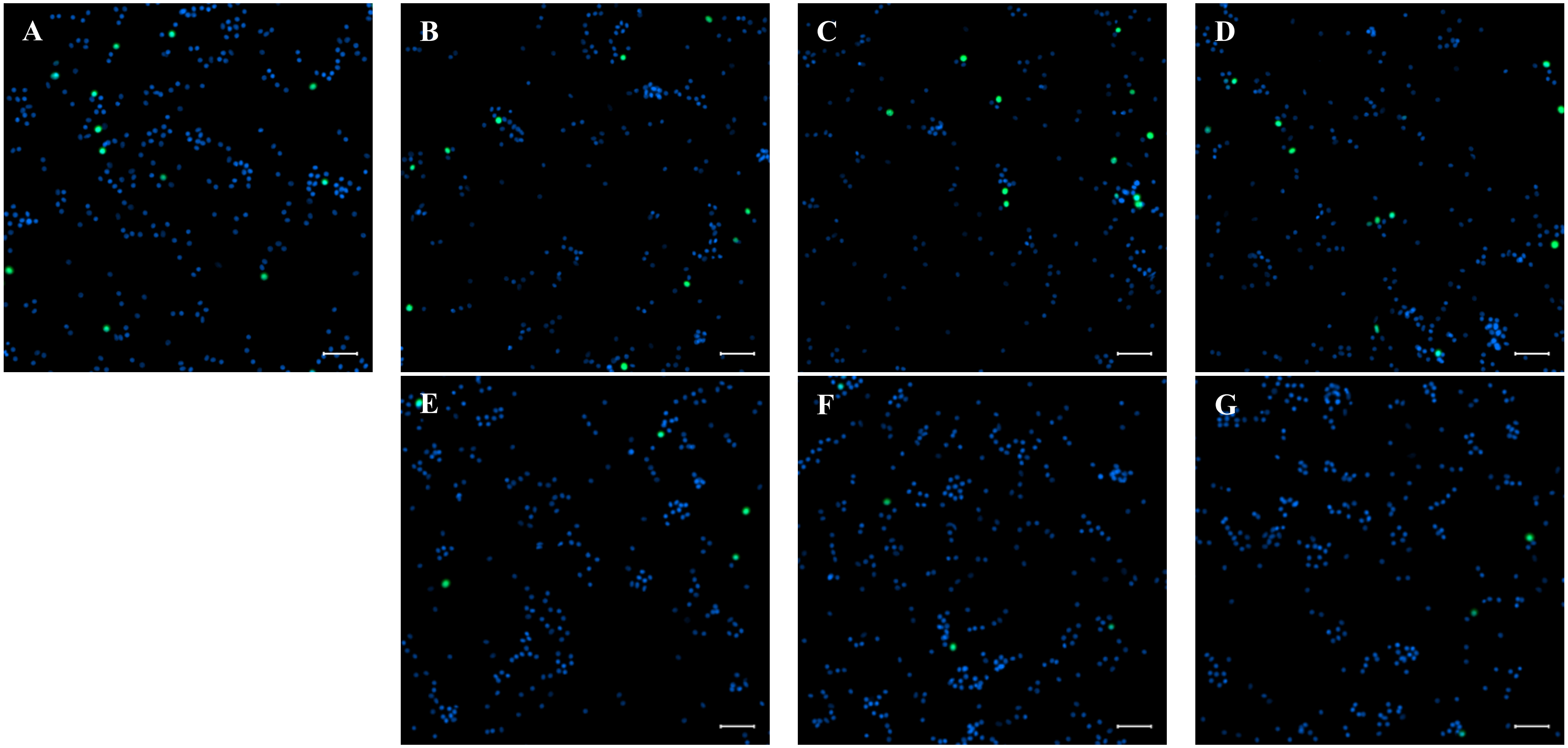

Figure S8. Representative images of EdU proliferation assays on chondrocytes treated with Sr-polyP. Primary bovine chondrocytes were treated with A) no particles as the control, Sr-polyP as synthesized at a concentration of B) 25 μg mL^{-1}, C) 50 μg mL^{-1}, and D) 100 μg mL^{-1} respectively, or sonicated Sr-polyP at a concentration of E) 25 μg mL^{-1}, F) 50 μg mL^{-1}, and G) 100 μg mL^{-1} respectively for 24 hours. Proliferating cells were labelled with an Alexa Fluor 488 azide (green) and nuclei were stained with Hoechst 33342 (blue). Scale bar = 50 μm.

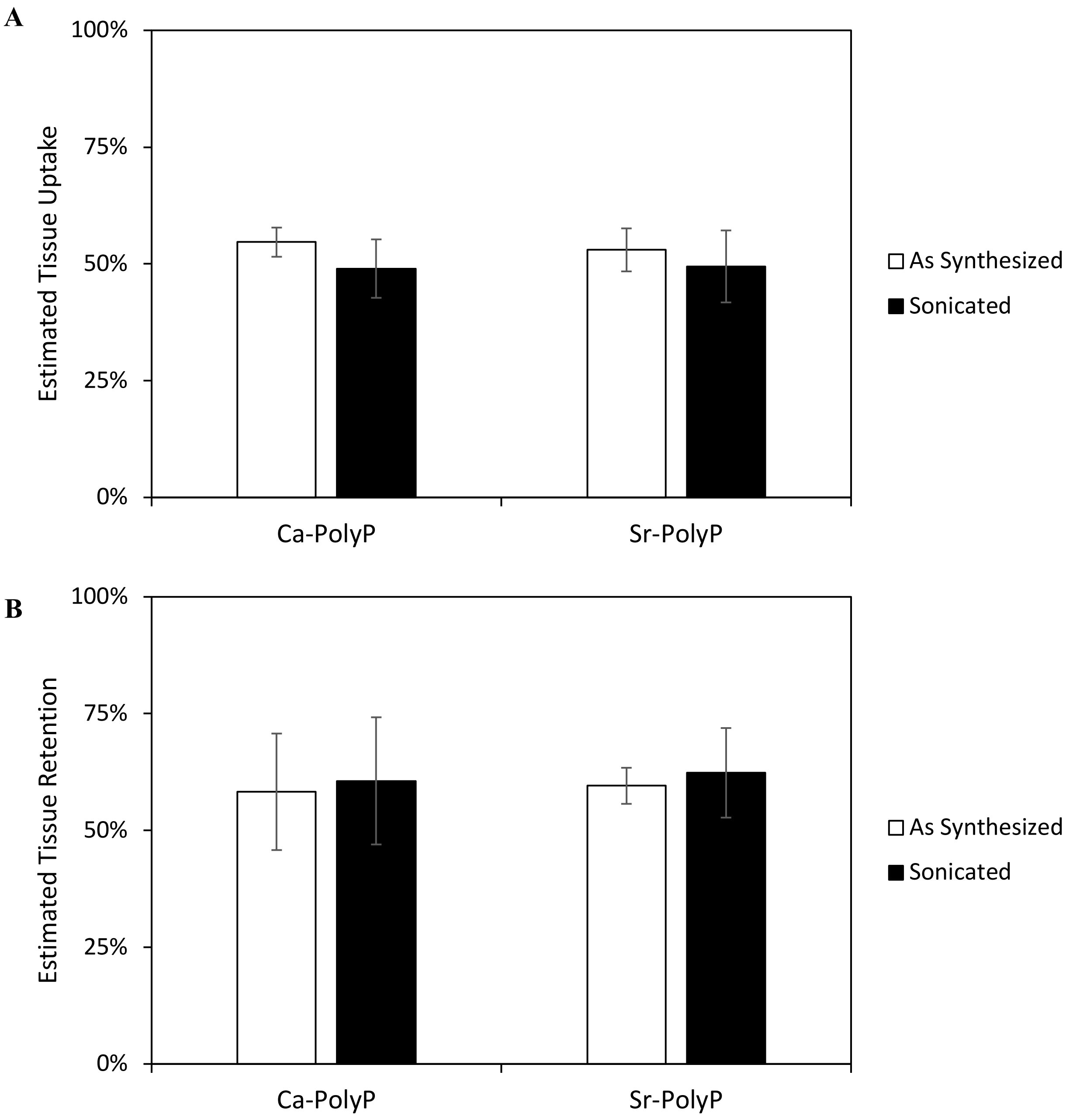

Figure S9. Quantification of particle penetration and retention in cartilage tissue explants. Bovine cartilage explants punched into 6 mm diameter discs were incubated with 100 μg mL^{-1} of DAPI-stained particles for 3 days on an orbital rocker at 37°C. A) Quantification of fluorescence decrease in solution as a measure of particle uptake into the cartilage tissue, compared to the initial solution. B) Cartilage explants were then subsequently incubated in the absence of particles and fluorescence in solution was quantified after an additional 3 days as a measure of particle release and compared to the amount of uptake prior to washing to determine particle retention within the cartilage tissue. Data are presented as averages ± standard deviation for $n \geq 3$ biological replicates with tissues isolated from different animals.
